# The Potential Role of Gut Microbiome Dysbiosis and Functional Reprogramming in Colorectal Liver Metastasis via the Gut–Liver Axis

**DOI:** 10.1002/cnr2.70738

**Published:** 2026-11-04

**Authors:** Yuejin Sheng, Xiaoyu Dai, Jiaqi Lu, Hao Zhang, Ping Cai

**Affiliations:** ^1^ Health Science Center, Ningbo University Ningbo Zhejiang China; ^2^ Xiangshan Hospital of TCM Medical and Health Group Ningbo Zhejiang China; ^3^ Ningbo No.2 Hospital Ningbo Zhejiang China; ^4^ School of Marine Sciences, Ningbo University Ningbo Zhejiang China

**Keywords:** biomarkers, colorectal liver metastasis, gut microbiome, gut‐liver axis, metagenomics, non‐invasive diagnosis

## Abstract

**Background:**

Colorectal liver metastasis (CRLM) is a leading cause of mortality in colorectal cancer (CRC), yet current surveillance often misses occult micrometastases, underscoring an urgent need for early non‐invasive biomarkers and mechanistic insights.

**Aims:**

This study aimed to investigate gut microbiota dysbiosis and functional alterations associated with CRLM and explore potential non‐invasive biomarkers.

**Methods and Results:**

Fecal metagenomic sequencing was performed in 60 participants, comprising healthy controls, patients with non‐metastatic CRC, and patients with synchronous CRLM (*n* = 20 per group). Microbial composition, functional pathways, co‐occurrence networks, and clinical associations were systematically analyzed, and a random forest model was constructed to identify potential diagnostic biomarkers. Results showed that gut microbiota dysbiosis intensified as CRC progressed toward CRLM, characterized by the depletion of commensal flora and significant enrichment of specific genera, such as *Eikenella* and *Porphyromonas*. Functionally, the gut microbiome displayed stage‐specific remodeling, shifting from infection‐ and virulence‐related pathways in CRC to functions associated with microbial secondary metabolism and stress adaptation in CRLM. Furthermore, SPIEC‐EASI‐based network analysis revealed a sparser and more compartmentalized microbial ecological structure in CRLM, with *[Clostridium] symbiosum*, *Campylobacter concisus*, and *Coprococcus eutactus* emerging as module hubs. A random forest model integrating nine core taxa and one virulence factor (VF0160 *FeoAB*) achieved an overall 97.5% classification accuracy in discriminating CRLM from non‐metastatic CRC, indicating the potential of fecal metagenomic biomarkers for future risk stratification. However, prospective validation in larger multicenter cohorts is required before clinical translational utility.

**Conclusion:**

Collectively, our findings suggest that gut microbiota dysbiosis and functional reprogramming are strongly associated with CRLM progression, potentially through remodeling the hepatic pre‐metastatic niche via the gut‐liver axis.

**Abbreviations**
BMI: body mass index
CEA: carcinoembryonic antigen
CRC: colorectal cancer
CRLM: colorectal liver metastasis
CRP: C‐reactive protein
FDR: false discovery rate
KEGG: Kyoto Encyclopedia of Genes and Genomes
MDA: mean decrease in accuracy
MRI: magnetic resonance imaging
NEU: neutrophil
ORF: open reading frame
PCoA: principal coordinate analysis
PERMANOVA: permutational multivariate analysis of variance
RF: random forest
TME: tumor microenvironment
TNM: Tumor‐Node‐Metastasis
VF: virulence factor
WBC: white blood cell

## 1 Introduction

Colorectal cancer (CRC) is the third most commonly diagnosed malignancy, which ranks as the second leading cause of mortality globally [1]. With global incidence projected to rise from 1.93 million in 2020 to 3.2 million by 2040, the clinical challenge posed by CRC is escalating [2, 3]. Mortality in CRC is predominantly driven by metastatic dissemination, with the liver as the most common site of distant metastasis. Approximately 30%–50% of CRC patients develop colorectal liver metastasis (CRLM), causing a steep decline in 5‐year survival rates from 90% in localized disease to just 13% in metastatic cases [4, 5]. The poor prognosis is largely attributed to the clinically silent nature of early dissemination. Current surveillance strategies often fail to detect occult micrometastases, leading to diagnosis at advanced stages. Consequently, there is an urgent need to elucidate the potential mechanisms driving CRLM and to identify robust, non‐invasive biomarkers that enable earlier risk stratification and therapeutic intervention.

The pathogenesis of CRLM is orchestrated by a complex interplay of genetic alterations, the gut microbiota, the tumor microenvironment, and environmental factors [6, 7]. Within this landscape, the gut microbiota has emerged as a pivotal modulator of tumor progression and metastasis [8]. Anchored by the gut–liver axis, intestinal microbes can remotely modulate the hepatic pre‐metastatic niche through the portal circulation, metabolite transport, and immune signaling [9, 10, 11, 12, 13]. Emerging evidence has shown that the gut microbiota exerts bidirectional effects within the gut–liver axis, potentially either promoting or suppressing CRLM [14, 15]. It has been reported that a few gut commensals, such as *Bacteroides vulgates* [11] and *Lactobacillus paracasei* [10], inhibit metastasis by enhancing immune surveillance. Conversely, pathobionts like *Fusobacterium nucleatum* [16, 17, 18] and *Escherichia coli* [19, 20, 21] not only accelerate the invasiveness of primary tumors but also persist within metastatic lesions [22, 23, 24]. Additionally, the composition of gut microbiota is significantly differed between patients with CRLM and those with non‐metastatic CRC [25]. These findings underscore the potential of microbiome features as non‐invasive biomarkers for early risk stratification [26]. However, given the high inter‐individual heterogeneity of the human fecal microbiome, it remains unclear whether additional high‐impact taxa are consistent drivers of CRLM. Furthermore, most existing studies have primarily focused on taxonomic alterations, with limited investigation into the functional mechanisms through which microbes promote the growth of metastatic lesions [11, 24].

A systematic understanding of how the gut microbiome drives the metastatic process at the functional level remains elusive. This knowledge gap significantly constrains both mechanistic insights and clinical translation. Mechanistically, microbial toxins create a biological milieu favorable for liver metastasis by dismantling intestinal barriers, inducing chronic inflammation, and remodeling the immune microenvironment [10, 27, 28]. Given the anatomical and immunological connections of the gut–liver axis, pathogenic toxins may further facilitate the establishment of a hepatic pre‐metastatic niche, thereby increasing the likelihood of tumor cell seeding and expansion in the liver [29, 30]. However, the specific functional pathways that play key roles in the progression from CRC to CRLM remain largely undefined [31]. Notably, compared with taxonomic composition, microbial functional modules are often more reproducible across individuals and more biologically interpretable, rendering them robust candidates for diagnostic biomarkers and therapeutic targets [32]. Therefore, refined investigations targeting microbial functions are urgently needed. Such studies aim to not only elucidate the potential mechanisms mediating CRLM but also evaluate their utility as non‐invasive molecular markers for early identification and risk stratification, thereby providing a basis for precise screening and intervention strategies.

To address aforementioned knowledge gaps, we conducted a comprehensive metagenomic analysis of fecal microbiome in a large cohort comprising healthy controls and patients with CRC or CRLM. We aimed to identify key microbial taxa and functions associated with CRLM and to infer their potential mechanisms. Specifically, rather than merely cataloging taxonomic shifts, we employed a multi‐dimensional analytical framework integrating functional pathway profiling, microbial co‐occurrence networks, and clinical correlations to infer the potential mechanisms during CRLM progression. Leveraging these coupled taxonomic and functional insights, we further developed a machine‐learning model for the early non‐invasive detection of CRLM. By delineating the associations between gut microbial composition/function and metastasis, this study lays the groundwork for microbiome‐based early diagnosis and timely intervention in CRLM.

## 2 Materials and Methods

### 2.1 Clinical Cohorts and Sample Collection

This study recruited 60 participants undergoing routine colonoscopy at Ningbo No.2 Hospital in 2024 and 2025. Patients were staged based on histopathological characteristics according to the Tumor‐Node‐Metastasis (TNM) staging system. Samples with uncertain TNM staging due to insufficient clinical evidence were excluded [33]. The cohort comprised three groups: non‐metastatic CRC (CRC, *n* = 20), synchronous CRC with liver metastasis (CRLM, *n* = 20; defined as metastases detected synchronously with or within 6 months of the primary tumor diagnosis), and healthy controls (CON, *n* = 20). Exclusion criteria were as follows: (1) patients with appendiceal tumors, familial adenomatous polyposis, anal canal tumors, or neuroendocrine tumors; (2) patients requiring additional surgery following endoscopic treatment due to complications such as perforation or bleeding; (3) patients who had received antibiotics, glucocorticoids, or immunosuppressants within 1 month prior to sampling [34]. Demographic and clinical metadata, including age, gender, body mass index (BMI), tumor type, tumor stage, and tumor site, were summarized in Table S1. Approximately 5 g of fresh fecal samples were collected from the central portion of each specimen, immediately flash‐frozen in liquid nitrogen, and transported to the laboratory within an icebox. Whole blood was collected in EDTA‐coated tubes for immediate hematological analysis. Serum samples were collected in serum separation tubes, allowed to clot at room temperature for 30 min, and centrifuged at 3000 rpm for 10 min to isolate serum for biochemical assays. Serum and fecal samples were stored at −80°C for subsequent analysis. This study was approved by the Ethics Committee of Ningbo No.2 Hospital (Approval No. PJ‐NBEY‐KY‐2024‐203‐01), and written informed consent was obtained from all participants.

### 2.2 Clinical Biochemistry

Serum levels of carcinoembryonic antigen (CEA) and C‐reactive protein (CRP) were quantified using the Ortho VITROS 5600 blood biochemical analyzer (Ortho Clinical Diagnostics, NJ, USA). White blood cell (WBC) and neutrophil (NEU) counts were determined in whole blood samples using the Sysmex XN‐9000 hematology analyzer (Sysmex Corporation, Kobe, Japan).

### 2.3 DNA Extraction and Metagenomic Sequencing

Total genomic DNA was extracted from fecal samples using the QIAamp Fast DNA Stool Mini Kit (Qiagen, Hilden, Germany) in accordance with the manufacturer's instructions. DNA concentration and purity were determined using a Qubit 4.0 Fluorometer (Life Technologies, CA, USA) and a NanoDrop Spectrophotometer (Thermo Fisher Scientific, MA, USA), respectively. High‐quality DNA samples were used to construct metagenomic sequencing libraries with the NEBNext Ultra DNA Library Prep Kit (New England Biolabs, Ipswich, MA, USA). Sequencing was performed on the Illumina NovaSeq 6000 platform (Magigene Biotechnology, Guangzhou, China) to generate 150 bp paired‐end reads. All raw data have been deposited in the GSA database under accession number CRA041604.

### 2.4 Bioinformatics Processing and Taxonomic Profiling

Raw reads underwent quality control using FastQC (v0.11.6) [35] and Trimmomatic (v0.38) [36] to remove adaptors and low‐quality reads (length < 36 bp, average quality score < 20). Host contamination was removed by mapping clean reads to the human reference genome (hg38) using BMTagger (v3.101) [37]. Following quality control and removal of human‐derived reads, the mean clean sequence yields were 6.35 ± 0.33, 6.15 ± 0.36, and 6.55 ± 0.35 Gb per sample for the CON, CRC, and CRLM groups, respectively (mean ± SD; Table S2). Taxonomic classification was subsequently performed using Kraken2 (v2.1.2) [38] against a reference database based on GTDB release 226 and supplemented with the hvfc subset of NCBI RefSeq. Bracken (v2.5) [39] was employed to re‐estimate taxonomic abundances by redistributing reads from higher‐level classifications.

### 2.5 Functional Annotation

High‐quality reads were assembled into contigs using MEGAHIT (v1.2.9) [40]. Open reading frames (ORFs) within the contigs (> 200 bp) were predicted using Prodigal (v2.6.3) [41]. A non‐redundant gene catalog was constructed using CD‐HIT (v4.8.1) [42] with a sequence identity threshold of 95% and alignment coverage of 90%. Gene abundances were quantified using Salmon (v0.11.3) [43] and normalized as transcripts per million (TPM). Functional annotation was performed by aligning ORFs from the gene catalog against the Kyoto Encyclopedia of Genes and Genomes (KEGG) database (https://www.genome.jp/kegg/genes.html) using DIAMOND (v0.9.18) [44] with an e‐value threshold of 1e‐5.

### 2.6 Construction and Topological Analysis of Microbial Co‐Occurrence Network

To explore interactions among microbial taxa, co‐occurrence networks were constructed using SPIEC‐EASI based on species‐level abundance profiles. Network inference was performed separately for the CON, CRC, and CRLM groups using sparse inverse covariance estimation, and the network sparsity was determined using the Stability Approach to Regularization Selection (StARS). Associations retained in the final sparse graphical models were used to construct the microbial ecological networks. Topological properties, including the number of nodes, number of edges, average degree, network density, distance, betweenness centrality, modularity, network efficiency, and stability, were extracted to quantitatively assess the structural complexity of the networks. To comprehensively assess microecological stability, we evaluated network robustness. Specifically, robustness was calculated by simulating species degradation (e.g., random or targeted removal of hub nodes) to measure the community's resistance to external perturbations [45]. Furthermore, the topological roles of nodes were classified based on their within‐module connectivity ($Z_{i}$) and among‐module connectivity ($P_{i}$) to identify potential keystone taxa maintaining the network architecture. According to established criteria, nodes were classified into peripherals, connectors ($P_{i}$ > 0.62), module hubs ($Z_{i}$ > 2.5), and network hubs ($Z_{i}$ > 2.5 and $P_{i}$ > 0.62). The latter three categories were collectively designated as potential keystone taxa [46]. The microbial co‐occurrence network was visualized using Gephi (v0.10.1) [47].

### 2.7 Construction of Diagnostic Model of CRLM

Random forest (RF) model was deployed to evaluate the diagnostic potential of the gut microbiota for CRLM using the random Forest package [48]. To minimize demographic bias, microbial genera and functional features significantly associated with age or sex in the healthy group were excluded prior to modeling [49]. Models were constructed using the filtered abundance profiles and functional profiles. To minimize overfitting, model development and evaluation were performed using a nested cross‐validation framework. In the outer cross‐validation loop, samples were stratified into training and held‐out test folds. Within each outer training set, feature importance was evaluated using mean decrease in accuracy (MDA) based on permutation testing [50], and feature selection and model optimization were performed exclusively within the inner cross‐validation loop. The optimized model was then applied to the corresponding outer test fold, which remained completely independent of feature selection and model training. Out‐of‐fold predictions from all outer test folds were aggregated to evaluate classification performance.

### 2.8 Statistical Analysis

Statistical analyses and visualizations were performed in R (v4.3.0), unless otherwise stated. Demographic and clinical variables among the three health statuses were compared using one‐way analysis of variance (ANOVA). Taxonomic and functional diversities were calculated using the vegan package. Group differences were assessed using the Kruskal–Wallis test, followed by the Wilcoxon rank‐sum test for pairwise comparisons. To account for potential demographic confounding, multivariable differential abundance analyses of microbial taxa and functional features were performed using MaAsLin2, with disease status as the primary explanatory variable and age and BMI included as continuous covariates. *p* values were adjusted using the Benjamini–Hochberg method to control the false discovery rate (FDR). Spearman's rank correlation analysis was performed to evaluate the associations between clinical variables and genus‐level microbial taxa as well as microbial functional pathways. All statistical tests were two‐tailed, with *p* < 0.05 considered statistically significant. Beta‐diversity was analyzed via principal coordinate analysis (PCoA) based on Bray–Curtis distances using the vegan and ggplot2 packages to visualize compositional dissimilarities in taxonomic and functional profiles among groups. Statistical differences were quantified using permutational multivariate analysis of variance (PERMANOVA) [51] with the adonis2 function (999 permutations). Mantel tests were conducted to assess the correlations between clinical characteristics and overall microbial community structure or microbial functional profiles using the vegan package.

## 3 Results

### 3.1 Clinical Biochemical Characteristics

Clinical biochemical indices varied significantly across the CON, CRC, and CRLM groups (*p* < 0.05, Figure 1). Levels of CEA, WBC, NEU, and CRP were significantly elevated in the CRLM group compared with the CRC and CON groups and were also significantly higher in the CRC group than in the CON group (*p* < 0.05). All above clinical variables showed a progressive increase along with the disease progression from healthy status to primary CRC and to CRLM.

**FIGURE 1:**
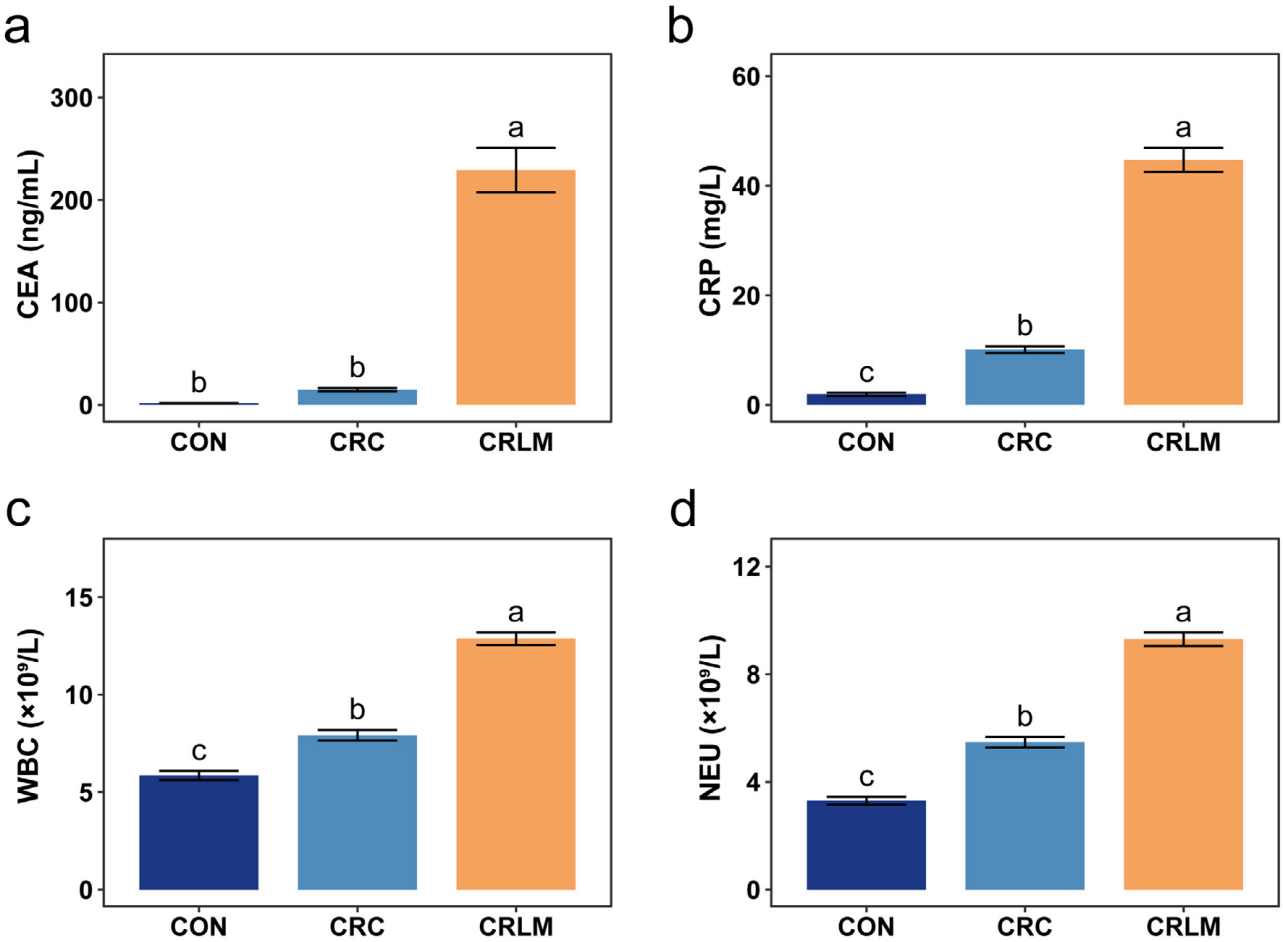
Comparison of clinical biochemistry among CON, CRC, and CRLM groups. Boxplots illustrating the levels of CEA, WBC, NEU, and CRP among three groups (a–d). CEA: carcinoembryonic antigen; CON: healthy controls; CRC: colorectal cancer; CRLM: colorectal liver metastasis; CRP: C‐reactive protein; NEU: neutrophil; WBC: white blood cell.

### 3.2 Gut Microbiota Dysbiosis in CRC and CRLM

To elucidate the microbial alterations associated with CRC and CRLM, we compared the structures of gut microbiota across CON, CRC, and CRLM groups. At the phylum level, Bacteroidota, Bacillota, and Pseudomonadota were the dominant phyla of the three groups (Figure 2a). Relative abundances of Bacteroidota and Bacillota were significantly elevated (*p* < 0.05), whereas Pseudomonadota was depleted in the CRC and CRLM groups compared with the CON group. Notably, the relative abundance of Fusobacteriota enriched (*p* < 0.05) in the disease groups, particularly in the CRLM group. While alpha‐diversity, as measured by the Shannon index, was comparable among the groups (Figure 2b), beta‐diversity analysis revealed significant differences in overall microbial community structure among the three groups (*p* = 0.002; PERMANOVA, permutations 999) (Figure 2c). Pairwise comparisons further indicated that these differences were primarily driven by comparisons involving the CRLM group, including comparisons between CON and CRLM and between CRC and CRLM, whereas no significant separation was observed between CON and CRC groups (Table S3). At the genus level, distinct dysbiotic signatures emerged in the disease groups (Figure 2d). Specifically, *Escherichia*, *Staphylococcus*, *Corynebacterium*, and *Phocaeicola* showed marked enrichment in the primary CRC group relative to the CON group (*p* < 0.05), suggesting a potential association with the primary tumor microenvironment. In contrast, with disease progression, the relative abundances of *Eikenella* and *Porphyromonas* were markedly enriched (*p* < 0.05) in the CRLM group. Conversely, *Neisseria*, *Finegoldia, Anaerococcus*, and *Roseburia* genera were significantly decreased in the CRLM group (*p* < 0.05).

**FIGURE 2:**
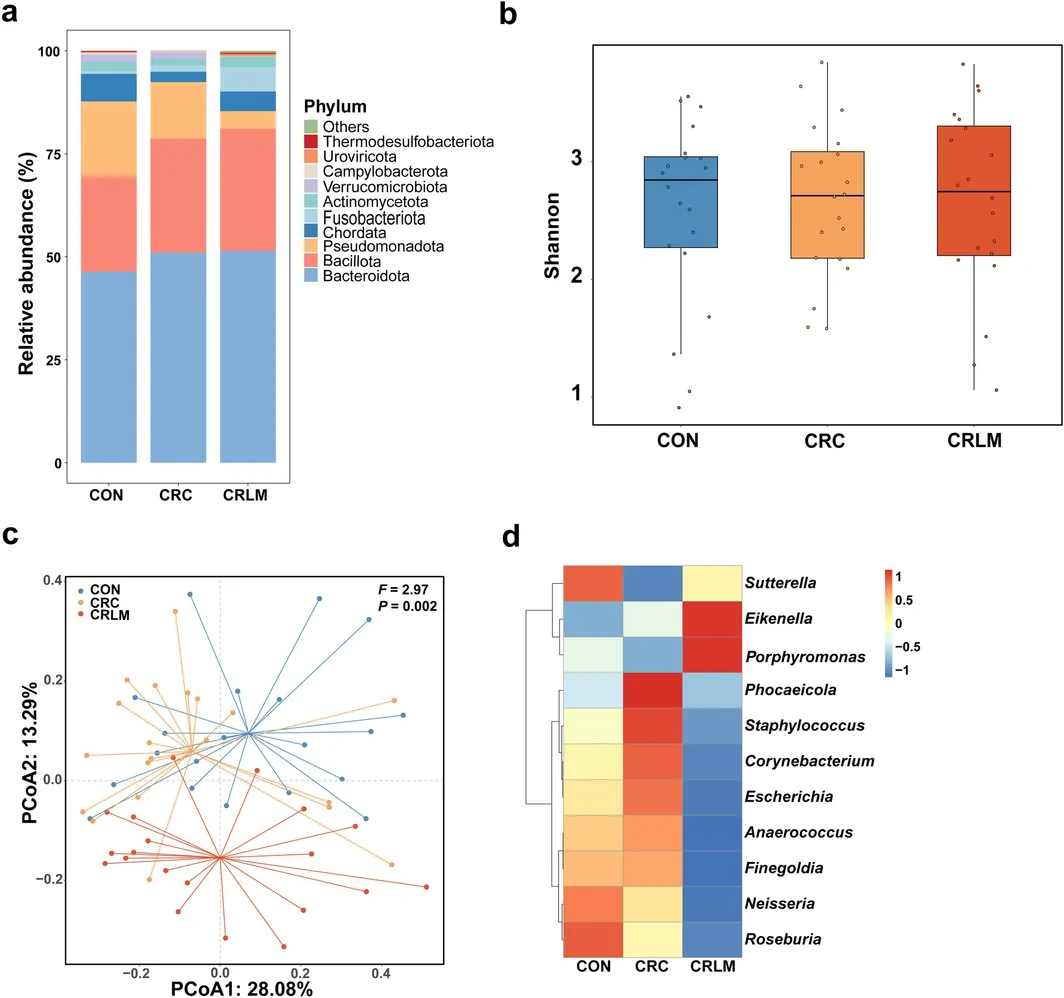
Microbial community composition and structural shifts in the CON, CRC, and CRLM groups. Stacked bar chart displaying the relative abundance (%) of the main bacterial phyla identified in different groups (a). Comparison of alpha‐diversity (Shannon index) among groups using the Kruskal–Wallis test (b). PCoA plots showing the differences in microbial composition among different groups based on Bray–Curtis distance (c). Heatmap depicting the differences in relative abundance of microbial genera among different groups (d). Refer Figure 1 for abbreviations.

### 3.3 Altered Functional Features of the Gut Microbiome in CRC and CRLM

Functional alpha‐diversity was largely comparable among groups (*p* > 0.05) (Figure 3a). PCoA revealed a statistically significant separation among the CON, CRC, and CRLM groups (*p* = 0.005; PERMANOVA, permutations 999) (Figure 3b). The heatmap revealed distinct pathway‐level alterations between the healthy and diseased groups (Figure 3c), with quantitative comparisons of pathway abundances summarized in Table S4. Compared with CON, pathways related to bacterial infection and virulence, including Virion—Bacteriophage lambda, Yersinia infection, Bacterial secretion system, and Lipopolysaccharide biosynthesis, were enriched in CRC (*p* < 0.05). Compared with the CON and CRC groups, the CRLM group exhibited a distinct metagenomic functional profile (Figure 3c). Several pathways that were enriched in the CRC group showed reduced representation in CRLM. In contrast, pathways associated with microbial secondary metabolism and stress adaptation, such as Biosynthesis of various antibiotics and Antifolate resistance, were enriched in the CRLM group (*p* < 0.05). These findings indicate a further remodeling of the gut microbial functional repertoire in association with liver metastasis.

**FIGURE 3:**
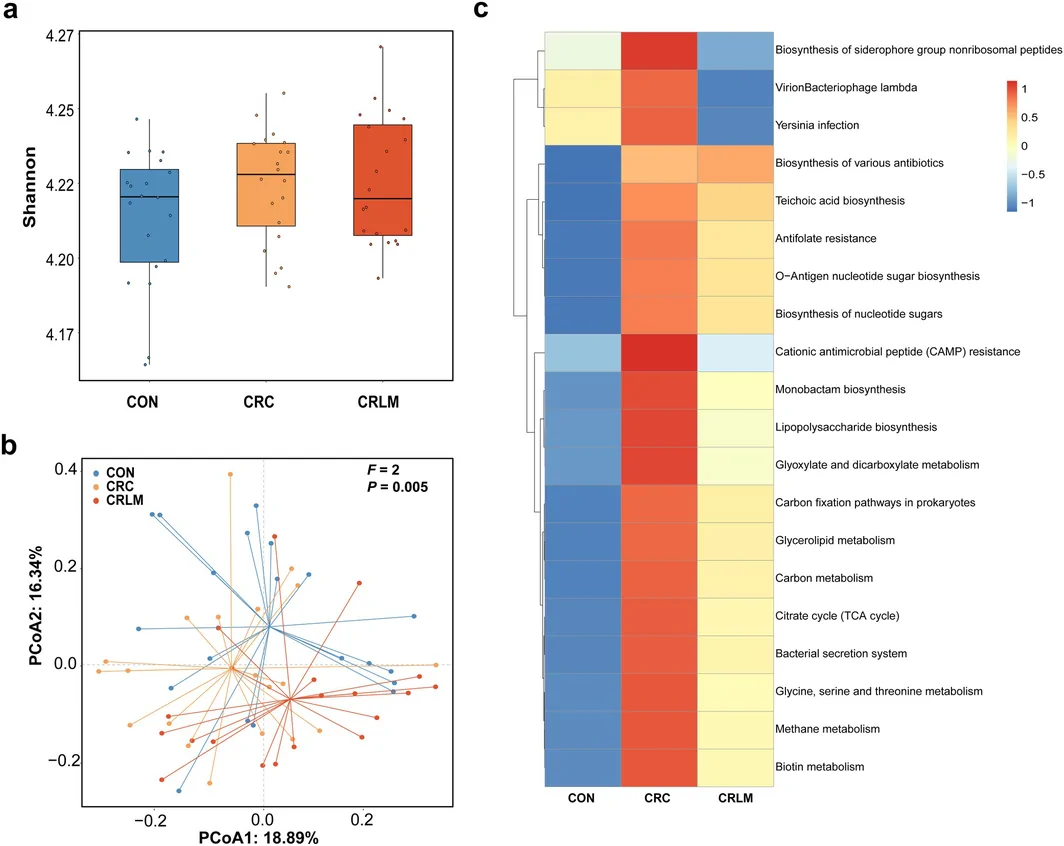
Comparison of the gut functional structures among CON, CRC, and CRLM groups. Comparison of alpha‐diversity (Shannon index) among groups using the Kruskal–Wallis test (a). PCoA plots showing the differences in microbial functional composition among groups based on Bray–Curtis distance (b). Heatmap showing differentially abundant functional pathways among different groups (c). Refer Figure 1 for abbreviations.

### 3.4 CRC Progression Alters the Co‐Occurrence Network Among Gut Commensals

Given that the significant dysbiosis induced by CRC progression may further disrupt the intricate web of microbial inter‐dependencies, we compared species‐level co‐occurrence networks among groups (Figure 4a). Visually, the clustering patterns of the networks changed markedly during the progression from the CON to CRC and CRLM. Quantitative analysis of topological properties further revealed significant inter‐group differences (Figure 4b). Although the total number of nodes did not differ significantly among the three groups, the CRLM network contained significantly fewer edges than the CRC network and exhibited a lower mean degree than both the CON and CRC networks (*p* < 0.05). Network density was also reduced in CRLM compared with CON, whereas the distance was significantly increased (*p* < 0.05). In addition, the CRLM network showed significantly higher modularity and lower network efficiency than both the CON and CRC networks (*p* < 0.05). In contrast, betweenness, robustness weight, and stability did not differ significantly among the three groups. Collectively, these results indicate that the CRLM‐associated microbial network was characterized by reduced connectivity and efficiency together with increased compartmentalization.

**FIGURE 4:**
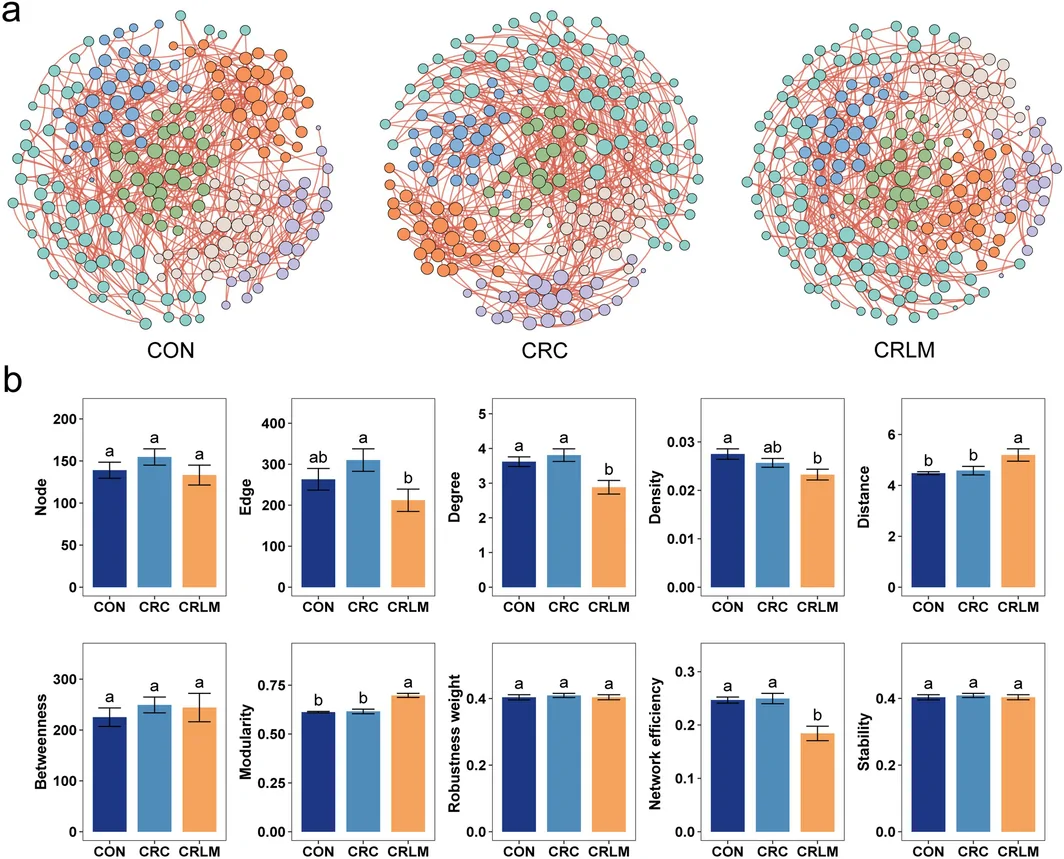
Comparison of gut microbial co‐occurrence networks in the CON, CRC, and CRLM groups. Microbial co‐occurrence networks constructed based on species‐level relative abundances in the three groups (a). Each node represents a microbial taxon, and each edge represents a significant correlation between taxa. Nodes with different colors indicate different network modules, and node size reflects the degree of connectivity within the network. Comparison of network topological properties among the three groups, including the number of nodes, number of edges, average degree, network density, distance, betweenness centrality, modularity, robustness weight, network efficiency, and stability (b). Refer Figure 1 for abbreviations.

$Z_{i}$‐$P_{i}$ analysis further revealed distinct shifts in the topological roles of microbial taxa across the three groups (Figure 5). In the CON network, most taxa were classified as peripheral nodes, accompanied by a smaller number of connectors, while *Bacteroides xylanisolvens* , *Mogibacterium diversum* , and *Fusobacterium pseudoperiodonticum* were identified as module hubs (Figure 5a, Table S5). At the CRC stage, the composition of topologically important taxa changed substantially, with multiple connector nodes and three module hubs, including *Phocaeicola salanitronis*, *Bacteroides* sp. A1C1, and *Odoribacter splanchnicus* (Figure 5b, Table S6). Upon progression to CRLM, peripheral nodes remained predominant, whereas the composition of both connectors and module hubs was further reorganized. Notably, *[Clostridium] symbiosum*, *Campylobacter concisus* , and *Coprococcus eutactus* emerged as module hubs in the CRLM network (Figure 5c, Table S7). No network hubs were identified in any of the three groups. Collectively, these results demonstrate a marked redistribution of taxa occupying key topological positions during progression from CRC to CRLM.

**FIGURE 5:**
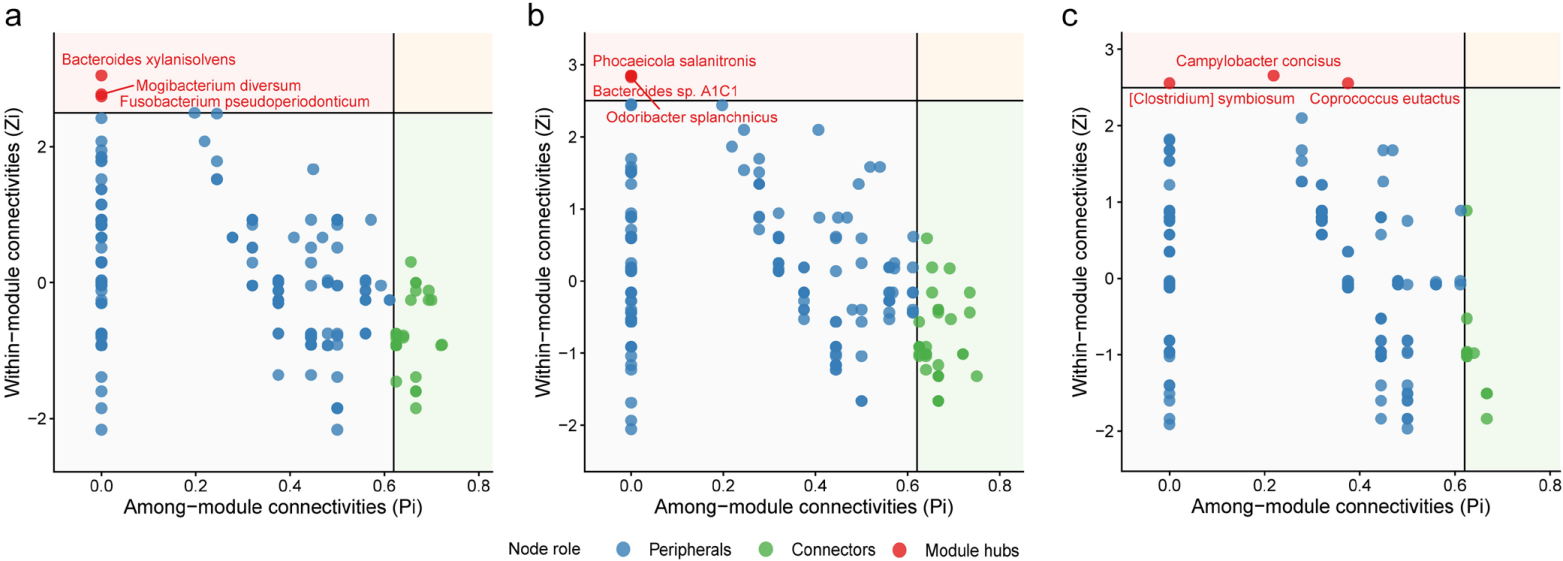
Topological roles of network nodes based on modularity analysis. The $Z_{i}$‐$P_{i}$ plot visualizes the classification of keystone species under distinct groups (a–c). Keystone taxa are categorized as module hubs ($Z_{i}$ > 2.5), connectors ($P_{i}$ > 0.62), or network hubs ($Z_{i}$ > 2.5 and $P_{i}$ > 0.62). Refer Figure 1 for abbreviations.

### 3.5 Correlation Between Characteristics of the Gut Microbiota and Host Clinical Parameters

To clarify the relationship between gut microecological alterations and host phenotypes, we evaluated the associations of specific microbial taxa and functions with clinical parameters. The Spearman correlation heatmap revealed that the relative abundances of multiple bacterial genera were significantly correlated with host clinicopathological and inflammatory indices (Figure 6a). Specifically, the commensal genera, including *Finegoldia, Neisseria*, *Anaerococcus*, *Staphylococcus*, and *Corynebacterium*, exhibited significant and negative correlations with tumor progression indicators of tumor stage, CEA, and host systemic inflammatory markers of WBC, NEU, and CRP (*r* ranging from −0.523 to −0.324, *p* < 0.05). These results indicate that the depletion of protective commensals is closely associated with increased tumor burden and the deterioration of the host's systemic inflammatory microenvironment.

**FIGURE 6:**
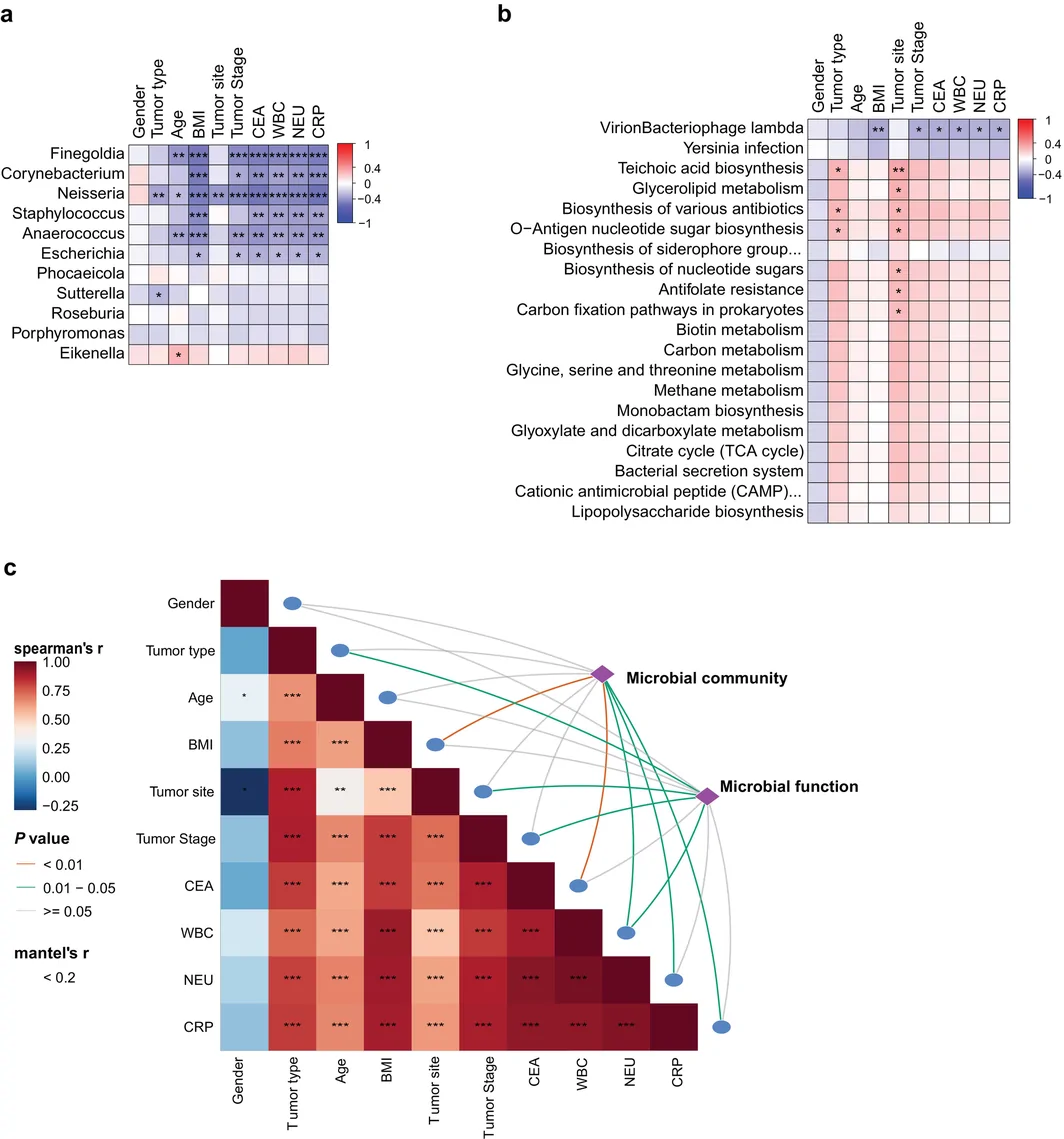
Interrelationship between microbial community composition or function and clinical indicators. Spearman correlations of specific microbial species and functional pathways with clinical parameters across different groups (a and b). Statistical significance is denoted by asterisks: *p < 0.05, **p < 0.01, and ***p < 0.001. Relationships between community structures (based on Bray–Curtis distance) and clinical variables using Mantel tests (c). Edge width is proportional to Mantel's *r* value, while edge color indicates statistical significance. Pairwise correlations among clinical variables are displayed via a color gradient representing Spearman's correlation coefficients. Refer Figure 1 for abbreviations.

The functional potentials of the gut microbiota also exhibited intimate synergy with clinical indices (Figure 6b). Specifically, infection‐associated pathways prominent in the earlier disease stages, such as Virion‐Bacteriophage lambda, showed significant and negative correlations with advanced tumor progression indicators (tumor stage and CEA) and systemic inflammatory markers (WBC, NEU, and CRP) (*r* ranging from −0.319 to −0.295, *p* < 0.05). Conversely, pathways associated with microbial secondary metabolism and stress adaptation, notably the Biosynthesis of various antibiotics and Antifolate resistance, displayed positive correlations with indicators of tumor progression (e.g., tumor type and tumor site) (*r* ranging from 0.261 to 0.275, *p* < 0.05). Taken together, these correlations indicate that the functional reprogramming of the gut microbiota is tightly coupled with the host's clinical trajectory.

The Mantel test demonstrated that the compositional and functional structure of the gut microbial community was closely associated with the host's systemic inflammatory status and indicators of tumor progression (Figure 6c). Specifically, the microbial composition was significantly associated with CEA, WBC, NEU, and CRP (Mantel's *r* ranging from 0.075 to 0.135, *p* < 0.05), whereas the microbial function was significantly associated with tumor type, tumor site, tumor stage, and WBC (Mantel's *r* ranging from 0.073 to 0.092, *p* < 0.05). Additionally, the pairwise clinical variable correlation matrix confirmed that inflammatory biochemical markers were significantly and positively correlated with tumor progression indicators (*r* ranging from 0.486 to 0.959, *p* < 0.001). These findings underscore a tightly intertwined relationship between the gut microbiome, human systemic inflammation, and tumor advancement.

### 3.6 Construction Non‐Invasive Model for Diagnosing CRLM

To improve the diagnostic efficacy for CRC and CRLM, we identified biomarkers based on single‐dimensional (microbiome or virulence factors alone) and multi‐dimensional (microbiome combined with virulence factors) profiles using the RF model. The results demonstrated that the multi‐dimensional model achieved higher diagnostic accuracy than microbiome or virulence factors alone (Figure 7; Figures S1–S8). In the most clinically challenging discrimination between CRLM and CRC, the multi‐dimensional model demonstrated promising classification performance within our single‐center discovery cohort. According to the feature importance ranking based on the MDA metric, the top 10 important features consisted of nine genus‐level microbial taxa and one virulence factor, with *Staphylococcus*, *Corynebacterium*, and *Finegoldia* contributing most significantly to the classification (Figure 7a). In addition, the virulence factor VF0160 *FeoAB* ranked prominently among these key features, representing a metagenomic functional signal derived from the total microbial gene pool. However, the intermediate contig‐level assembly files required for retrospective taxonomic binning could not be retrieved, precluding definitive attribution of *FeoAB*‐encoding sequences to specific bacterial species. Notably, some top‐ranked features did not exhibit significant univariate differences (Table S8). However, RF feature selection via MDA can capture non‐linear feature interactions and multi‐variable conditional dependencies across the entire feature space, enabling variables without significant univariate differences to contribute meaningfully to multi‐marker multivariate classification ensembles. The cross‐validation error curve further confirmed that the model effectively minimized classification error during feature selection (reaching a minimum error of 0.081). Using 0.50 as the predictive probability threshold, the multi‐dimensional model effectively distinguished CRLM samples from CRC samples, with CRLM samples generally showing predicted values > 0.50 and CRC samples generally showing predicted values < 0.50, achieving an overall predictive accuracy of 97.5% (Figure 7b). Taken together, these findings highlight the potential of integrating microbial taxa and virulence factors for distinguishing CRLM from non‐metastatic CRC, although further validation in independent and large cohorts is required to establish the generalizability of the model.

**FIGURE 7:**
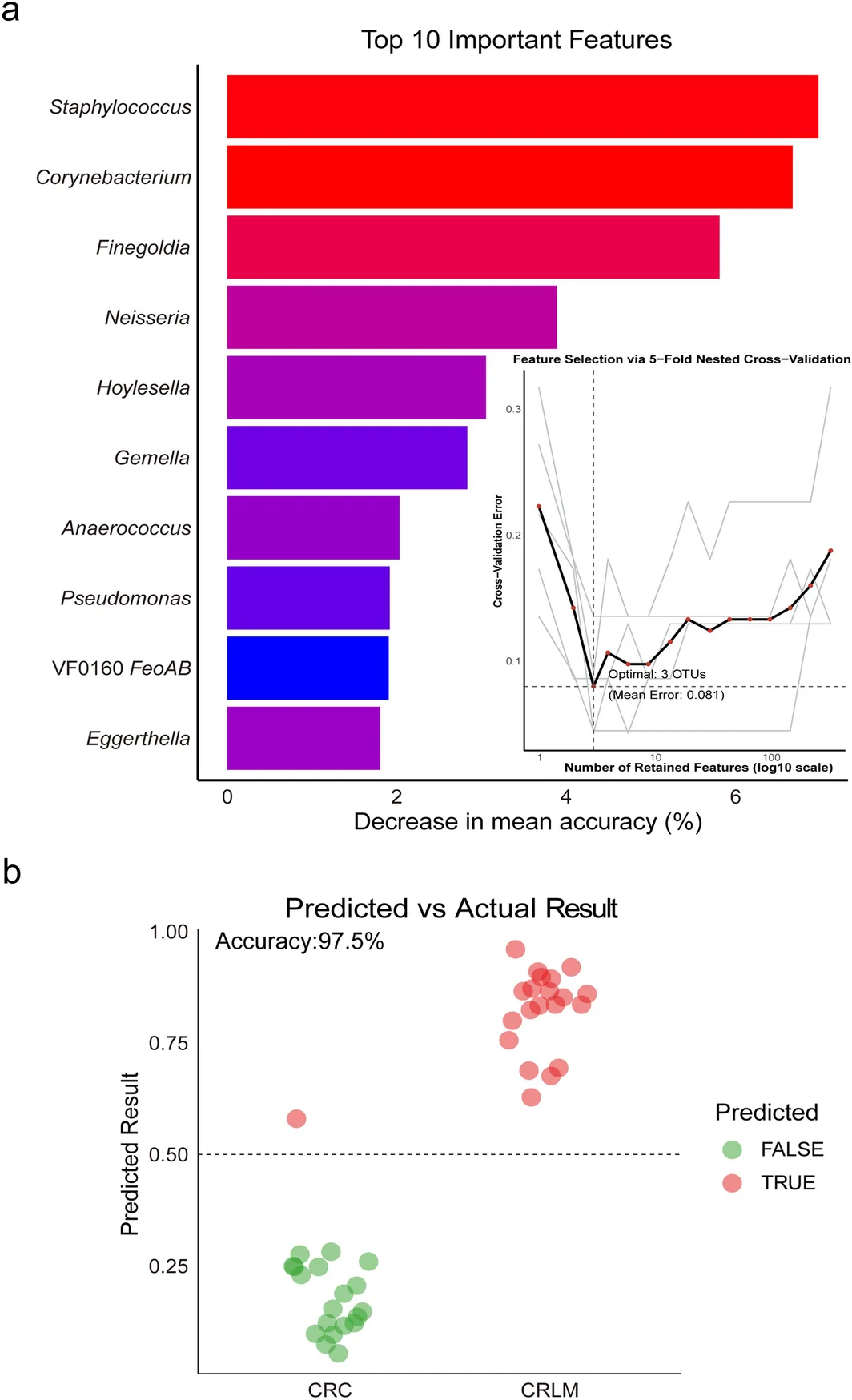
RF model integrating microbiome and virulence factors for distinguishing CRLM from CRC. The horizontal bar plot displays the Top 10 features ranked by the percentage of MDA (%). The inset illustrates the feature selection process based on nested cross‐validation, showing the variation in inner cross‐validation error relative to the number of features, with the dashed line indicating the point of minimum error (a). The scatter plot illustrates the predicted probabilities of the RF model for the samples (b). The horizontal dashed line denotes the classification threshold at a predicted result of 0.50. Data points are color‐coded according to the predicted category: red (TRUE) represents a prediction of CRLM, while green (FALSE) represents a prediction of CRC. The model achieved an overall classification accuracy of 97.5%.

## 4 Discussion

CRLM represents the leading cause of disease progression and mortality among patients with CRC [31]. Consequently, elucidating the underlying mechanisms of CRLM pathogenesis and establishing early detection approaches constitute critical unmet clinical needs in current CRC management [52, 53, 54]. Although recent studies have established the pivotal role of gut‐liver axis‐mediated gut dysbiosis in the progression of CRLM, current research predominantly focuses on phenotypic characterizations of microbial community composition [25, 31]. Accurately identifying the keystone microbial taxa associated with CRLM from within complex microbial networks and unraveling the specific functional pathways that potentially contribute to metastasis remain pressing bottlenecks that urgently require resolution [55]. To address this challenge, the present study established a stepwise progression cohort comprising healthy controls, non‐metastatic CRC patients, and patients with synchronous CRLM. Utilizing depth fecal metagenomic sequencing, we integrated pathway enrichment, ecological network analysis, and machine learning algorithms to systematically delineate the microbial dynamics along the progression of CRLM. This research not only elucidates the compositional, ecological, and functional alterations of the gut microbiota during CRLM progression but also identifies potential keystone nodes within the CRLM microbial network. Furthermore, it reveals potential mechanisms underlying microbiota‐mediated metastasis and develops a machine‐learning model for the non‐invasive discrimination of CRLM from non‐metastatic CRC.

At the taxonomic level, significant alterations in microbial community structure were primarily observed in comparisons involving CRLM patients (Figure 2c, Table S3), suggesting that microbiome remodeling is associated with liver metastatic dissemination. Notably, the abundance of the phylum Fusobacteriota was significantly enriched in the CRLM group (Figure 2a). This observation aligns with the widely accepted consensus that *Fusobacterium* species can translocate and colonize the liver alongside primary tumor cells, thereby sustaining a pro‐tumorigenic microenvironment at the metastatic site [18]. At the genus level, the CRC stage is predominantly characterized by the moderate disruption of the microecological environment (Figure 2d) and the localized colonization of pathogenic bacteria (e.g., *Escherichia*). Upon progression to the CRLM stage, commensal flora undergo further depletion, which is accompanied by a marked expansion of specific taxa (Figure 2d). These structural shifts may carry relevant biological implications for metastatic progression. In particular, depletion of butyrate‐producing commensals such as *Roseburia* has been associated with a compromised intestinal epithelial barrier and a reduction in colonization resistance [56, 57]. Concurrently, the enrichment of mucosa‐associated pathobionts such as *Eikenella* and *Porphyromonas* may contribute to local inflammation and immune perturbation [58, 59]. Consequently, we speculate that the progressive loss of protective commensals, together with the expansion of specific pro‐inflammatory pathobionts, may disrupt the intestinal barrier and, through the gut–liver axis, promote the establishment of a pre‐metastatic hepatic microenvironment characterized by inflammation and immunosuppression, thereby creating favorable conditions for metastatic colonization [10].

Accompanying the alterations in community structure, the functional potential of the gut microbiota also underwent profound remodeling. The CRC stage is primarily enriched in pathways associated with bacteriophages and infections (Figure 3c), reflecting the initial severe perturbation of the TME and the compromised physical barrier in host–microbe interactions [25, 55]. The enrichment of bacteriophage‐related pathways is consistent with findings regarding the regulation of CRC pathogenesis by the gut virome. Similarly, unique gut virome signatures are identified in patients with CRC, suggesting that the bacteriophage community may promote tumorigenesis either by exacerbating bacterial dysbiosis or by directly interacting with the host immune system [60]. Concurrently, the enrichment of the Yersinia infection pathway likely reflects an increase in genes encoding broad‐spectrum bacterial virulence factors, which facilitates bacterial invasion and the evasion of immune surveillance within the tumor microenvironment [61, 62]. In contrast, CRLM displayed a distinct functional profile, characterized by attenuation of several CRC‐enriched infection‐ and virulence‐related pathways and relative enrichment of functions associated with secondary metabolism and stress adaptation. These changes suggest a shift in microbial functional potential during metastatic disease. Coupled with elevated systemic inflammatory markers (e.g., CRP and WBC), these changes may reflect microbial adaptation to a more inflammatory and competitive ecological environment [63]. Such functional remodeling could enhance microbial persistence under adverse conditions and may contribute to maintaining a microenvironment favorable for metastatic colonization [10].

SPIEC‐EASI‐based microbial association network analysis revealed a marked reorganization of the gut microbial ecosystem during disease progression. Compared with the CRC network, the CRLM network exhibited reduced connectivity and network efficiency, together with increased path length and modularity, suggesting a sparser, less integrated, and more compartmentalized microbial community structure during metastatic disease [64, 65]. The succession of topological roles identified by $Z_{i}$‐$P_{i}$ analysis further supported this ecological reorganization. At the CRC stage, *Phocaeicola salanitronis*, *Bacteroides* sp. A1C1, and *Odoribacter splanchnicus* were identified as module hubs (Figure 5b). *O. splanchnicus* has previously been reported as a CRC‐associated keystone or driver taxon and was enriched in some CRC cohorts [66, 67]. Upon progression to CRLM, the composition of module hubs shifted markedly, with *[Clostridium] symbiosum*, *Campylobacter concisus* , and *Coprococcus eutactus* occupying central within‐module positions (Figure 5c). It has been reported that *C. symbiosum* directly implicates in colorectal tumorigenesis, promoting tumor progression via branched‐chain amino acid (BCAA) synthesis, host cholesterol metabolism alteration, and Hedgehog signaling pathway activation [68]. *C. concisus* is an opportunistic mucosal pathobiont known to co‐aggregate with *Fusobacterium nucleatum* , impair intestinal epithelial tight junction integrity, and trigger mucosal inflammatory cascades [69, 70]. Conversely, *C. eutactus* is a beneficial, short‐chain fatty acid (SCFA)‐producing commensal (specifically butyrate) [71]. This strain acted as a module hub within a sparser, highly modular network in CRLM (Figure 5c), suggesting that commensal butyrate producers become isolated within distinct, disconnected network sub‐modules as pathobionts expand. Together, these findings suggest that metastatic progression is accompanied by a redistribution of microbial ecological roles. Importantly, module‐hub status reflects topological importance rather than causality, and the specific roles of these taxa in CRLM require further functional validation.

Mantel tests and correlation analyses between the microbiota and clinical features provide valuable insights into the potential role of the “microbiome‐inflammation‐tumor” axis in clinical deterioration. We observed that taxa severely depleted during the CRLM stage exhibited strikingly synchronized negative correlations with host tumor burden and systemic inflammatory indices, whereas functional pathways associated with microbial secondary metabolism and stress adaptation showed positive correlations with tumor progression (Figure 6a,b). Notably, the taxonomic structure of the microbial community is predominantly associated with systemic inflammation, whereas microbial function is more intimately linked to the physical characteristics of the tumor itself (Figure 6c). This pattern suggests that the host's inflammatory state acts as both a catalyst for and a consequence of microecological dysbiosis, establishing a positive feedback loop that collectively accelerates the metastatic cascade [61, 72]. Specifically, we hypothesize that an increased tumor burden may contribute to systemic inflammation in the host. This inflammatory stress, together with the depletion of barrier‐maintaining commensals, may drive functional adaptation of the microbial community. Concurrently, impairment of the intestinal barrier may promote the translocation of gut‐derived microbial products to the liver through the gut‐liver axis. Such processes could further sustain systemic inflammation and contribute to the establishment of an immunosuppressive pre‐metastatic hepatic niche, thereby creating conditions favorable for metastatic colonization [10, 11, 63].

Currently, the clinical diagnosis of CRLM primarily relies on imaging modalities such as contrast‐enhanced abdominal and pelvic CT/MRI, alongside serum CEA testing [73, 74]. However, existing diagnostic approaches suffer from significant limitations, including delayed detection and insufficient sensitivity. Imaging examinations can only identify macroscopic metastatic lesions and fail to detect occult micrometastases [75]. Meanwhile, the sensitivity of CEA is merely 40%–60%, with nearly 30% of CRLM patients maintaining CEA levels within the normal range [76]. In contrast, our RF model, which integrates key microbial taxa and virulence factors (e.g., *Staphylococcus*, *Corynebacterium*, *Finegoldia*, and VF0160 *FeoAB*), achieved a 97.5% accuracy in distinguishing non‐metastatic CRC from CRLM (Figure 7b). Crucially, these predictive features are not merely statistical markers but may reflect underlying biological alterations. The decreased abundance of *Staphylococcus*, *Corynebacterium*, and *Finegoldia* may reflect disruption of commensal microbial homeostasis, leading to impaired intestinal barrier integrity and increased microbial translocation [15, 77]. VF0160 *FeoAB* confers microbial ferrous iron acquisition, which, alongside siderophore‐mediated ferric iron uptake, may support bacterial persistence through iron competition under heightened inflammation and oxidative stress in the dysbiotic gut associated with CRLM [78, 79]. However, these interpretations remain speculative, as unavailable contig‐level assembly files preclude species‐level attribution of *FeoAB*, and its biological activity was not directly assessed. Compared to current clinical tools, this model requires only a fecal sample, thus provides a non‐invasive, radiation‐free detection approach. This model holds potential to assist in the preoperative risk stratification of liver metastasis in CRC patients, whereas prospective validation in larger multicenter cohorts is required before clinical application.

This study provides a valuable foundation for understanding microbiota‐associated CRLM progression; however, we recognize some inherent limitations. First, the relatively limited sample size (*n* = 20 per group) may constrain the statistical power and generalizability of our findings. Although the machine learning model demonstrated promising discriminatory performance within the present single‐center cohort, prospective validation in larger multicenter cohorts is required to assess its generalizability and clinical utility. Second, detailed dietary information was not systematically collected in the present cohort, and potential dietary effects on gut microbiome composition and diversity therefore could not be fully assessed. Future prospective studies incorporating standardized dietary assessments are needed to better evaluate and control for this potential confounding factor. Third, consistent with the nature of metagenomic profiling, our findings primarily reveal biological associations rather than definitive causality. Microbiota‐derived metabolites, such as SCFAs, tissue‐level metastasis‐related markers, including EMT‐associated proteins, and inflammatory cytokines, such as IL‐1β and TNF‐α, were not directly assessed in the present study. Future studies integrating metagenomics with metabolomics, tumor tissue profiling, cytokine measurements, and in vivo and in vitro experiments are required to validate these associations and elucidate the underlying biological mechanisms.

## 5 Conclusions

This study provides a systematic and comprehensive characterization of the ecological succession, functional reprogramming, and co‐occurrence network dynamics of the gut microbiota during the progression from health to non‐metastatic CRC and, ultimately, to CRLM. Our findings indicate that CRLM is associated not only with shifts in microbial species abundance but also with a marked reorganization of the gut microbial ecological network, characterized by reduced connectivity and network efficiency together with increased modularity. This network restructuring was accompanied by a redistribution of taxa occupying key topological roles. In parallel, stage‐specific functional remodeling was observed, with non‐metastatic CRC showing infection‐ and virulence‐related microbial functions and CRLM displaying a distinct functional repertoire involving microbial secondary metabolism and stress adaptation. Based on these insights, we developed a multidimensional metagenomic classification model that integrates core microbial taxa with functional virulence factors. This model achieved an overall 97.5% accuracy in distinguishing CRLM from non‐metastatic CRC within the present cohort. In summary, this study provides further evidence that gut microbiome alterations are associated with CRLM and highlights their potential relevance to future risk stratification and microbiome‐based intervention strategies. Nevertheless, prospective validation in larger multicenter cohorts is required before clinical translational utility.

## Author Contributions

**Yuejin Sheng:** conceptualization, methodology, validation, formal analysis, investigation, visualization, writing – original draft. **Xiaoyu Dai:** methodology, validation, formal analysis, investigation, resources, data curation, writing – review and editing. **Jiaqi Lu:** visualization, writing – review and editing. **Hao Zhang:** writing – review and editing, supervision. **Ping Cai:** conceptualization, resources, writing – review and editing, supervision, project administration, funding acquisition.

## Funding

This work was supported by Medical Scientific Research Foundation of Zhejiang Province, China (2024KY1551, 2025KY1386, 2025KY1503) and the Science and Technology Innovation Yongjiang 2035 Key Research and Development Project of Ningbo (2024Z229), and Zhejiang Provincial Clinical Research Center for CANCER (2022E50008, 2024ZY01056).

## Ethics Statement

The study was conducted in accordance with the Declaration of Helsinki, and approved by the Ethics Committee of Ningbo No.2 Hospital (Approval No. PJ‐NBEY‐KY‐2024‐203‐01).

## Conflicts of Interest

The authors declare no conflicts of interest.

## Supporting information


**Figure S1:** Random Forest (RF) model based on microbiome for distinguishing CRC from CON.
**Figure S2:** RF model based on microbiome for distinguishing CRLM from CON.
**Figure S3:** RF model based on microbiome for distinguishing CRLM from CRC.
**Figure S4:** RF model based on virulence factors for distinguishing CRC from CON.
**Figure S5:** RF model based on virulence factors for distinguishing CRLM from CON.
**Figure S6:** RF model based on virulence factors for distinguishing CRLM from CRC.
**Figure S7:** RF model integrating microbiome and virulence factors for distinguishing CRC from CON.
**Figure S8:** RF model integrating microbiome and virulence factors for distinguishing CRLM from CON.
**Table S1:** Demographic and clinical characteristics in the CON, CRC, and CRLM groups.
**Table S2:** Raw and clean sequencing yields for individual samples.
**Table S3:** Pairwise Adonis (PERMANOVA) analysis of gut microbial community structure among the CON, CRC, and CRLM groups.
**Table S4:** Comparison of significantly different KEGG pathways among the CON, CRC, and CRLM groups.
**Table S5:** Module hubs and connectors in the CON microbial co‐occurrence network.
**Table S6:** Module hubs and connectors in the CRC microbial co‐occurrence network.
**Table S7:** Module hubs and connectors in the CRLM microbial co‐occurrence network.
**Table S8:** Abundance comparison of the top 10 features selected by the random forest model between the CRC and CRLM groups.

## Data Availability

The raw sequencing data generated in this study have been deposited in the Genome Sequence Archive (GSA) hosted by the National Genomics Data Center (NGDC), China National Center for Bioinformation (CNCB), with the accession numbers CRA041604. These data are publicly accessible via the GSA database (https://ngdc.cncb.ac.cn/gsa/). All other relevant data supporting the findings of this study are included in the article or available from the corresponding authors upon reasonable request.

## References

[1] Y. Xi and P. Xu , “Global Colorectal Cancer Burden in 2020 and Projections to 2040,” Translational Oncology 14, no. 10 (2021): 101174, 10.1016/j.tranon.2021.101174. PMC8273208 34243011

[2] E. Morgan , M. Arnold , A. Gini , et al., “Global Burden of Colorectal Cancer in 2020 and 2040: Incidence and Mortality Estimates From GLOBOCAN,” Gut 72, no. 2 (2023): 338–344, 10.1136/gutjnl-2022-327736. 36604116

[3] H. Sung , J. Ferlay , R. L. Siegel , et al., “Global Cancer Statistics 2020: GLOBOCAN Estimates of Incidence and Mortality Worldwide for 36 Cancers in 185 Countries,” CA: A Cancer Journal for Clinicians 71, no. 3 (2021): 209–249, 10.3322/caac.21660. 33538338

[4] A. Cervantes , R. Adam , S. Roselló , et al., “Metastatic Colorectal Cancer: ESMO Clinical Practice Guideline for Diagnosis, Treatment and Follow‐Up,” Annals of Oncology 34, no. 1 (2023): 10–32, 10.1016/j.annonc.2022.10.003. 36307056

[5] O. Bouche , T. Conroy , P. Michel , C. Penna , and C. Tournigand , “Metastatic Colorectal Cancer,” Gastroentérologie Clinique et Biologique 30, no. 2 (2006): 2S30–32S42. 17151561

[6] F. Bray , M. Laversanne , H. Sung , et al., “Global Cancer Statistics 2022: GLOBOCAN Estimates of Incidence and Mortality Worldwide for 36 Cancers in 185 Countries,” CA: A Cancer Journal for Clinicians 74, no. 3 (2024): 229–263, 10.3322/caac.21834. 38572751

[7] N. Keum and E. Giovannucci , “Global Burden of Colorectal Cancer: Emerging Trends, Risk Factors and Prevention Strategies,” Nature Reviews. Gastroenterology & Hepatology 16, no. 12 (2019): 713–732, 10.1038/s41575-019-0189-8. 31455888

[8] W. Cui , M. Guo , D. Liu , et al., “Gut Microbial Metabolite Facilitates Colorectal Cancer Development via Ferroptosis Inhibition,” Nature Cell Biology 26, no. 1 (2024): 124–137, 10.1038/s41556-023-01314-6. 38168770

[9] N. Yuan , X. Li , M. Wang , et al., “Gut Microbiota Alteration Influences Colorectal Cancer Metastasis to the Liver by Remodeling the Liver Immune Microenvironment,” Gut and Liver 16, no. 4 (2022): 575–588, 10.5009/gnl210177. PMC9289841 35318288

[10] A. Bertocchi , S. Carloni , P. S. Ravenda , et al., “Gut Vascular Barrier Impairment Leads to Intestinal Bacteria Dissemination and Colorectal Cancer Metastasis to Liver,” Cancer Cell 39, no. 5 (2021): 708–724.e11, 10.1016/j.ccell.2021.03.004. 33798472

[11] Q. L. Liu , H. Zhou , Z. Wang , and Y. Chen , “Exploring the Role of Gut Microbiota in Colorectal Liver Metastasis Through the Gut‐Liver Axis,” Frontiers in Cell and Developmental Biology 13 (2025): 1563184, 10.3389/fcell.2025.1563184. PMC11965903 40181829

[12] C. Ma , M. Han , B. Heinrich , et al., “Gut Microbiome–Mediated Bile Acid Metabolism Regulates Liver Cancer via NKT Cells,” Science 360, no. 6391 (2018): eaan5931, 10.1126/science.aan5931. PMC6407885 29798856

[13] S. Zeuzem , “Gut‐Liver Axis,” International Journal of Colorectal Disease 15, no. 2 (2000): 59–82, 10.1007/s003840050236. 10855547

[14] M. Rebersek , “Gut Microbiome and Its Role in Colorectal Cancer,” BMC Cancer 21 (2021): 1325, 10.1186/s12885-021-09054-2. PMC8666072 34895176

[15] P. Cai , Q. Yang , J. Lu , X. Dai , and J. Xiong , “Fecal Bacterial Biomarkers and Blood Biochemical Indicators as Potential Key Factors in the Development of Colorectal Cancer,” mSystems 10, no. 3 (2025): e00043‐25, 10.1128/msystems.00043-25. PMC11915818 40013832

[16] H. Yin , Z. Miao , L. Wang , et al., “ *Fusobacterium nucleatum* Promotes Liver Metastasis in Colorectal Cancer by Regulating the Hepatic Immune Niche and Altering Gut Microbiota,” Aging (Albany NY) 14, no. 4 (2022): 1941–1958, 10.18632/aging.203914. PMC8908934 35212644

[17] M. Zepeda‐Rivera , S. S. Minot , H. Bouzek , et al., “A Distinct *Fusobacterium nucleatum* Clade Dominates the Colorectal Cancer Niche,” Nature 628, no. 8007 (2024): 424–432, 10.1038/s41586-024-07182-w. PMC11006615 38509359

[18] S. Bullman , C. S. Pedamallu , E. Sicinska , et al., “Analysis of Fusobacterium Persistence and Antibiotic Response in Colorectal Cancer,” Science 358, no. 6369 (2017): 1443–1448, 10.1126/science.aal5240. PMC5823247 29170280

[19] A. Swidsinski , M. Khilkin , D. Kerjaschki , et al., “Association Between Intraepithelial *Escherichia coli* and Colorectal Cancer,” Gastroenterology 115, no. 2 (1998): 281–286, 10.1016/s0016-5085(98)70194-5. 9679033

[20] M. Bonnet , E. Buc , P. Sauvanet , et al., “Colonization of the Human Gut by *E. coli* and Colorectal Cancer Risk,” Clinical Cancer Research 20, no. 4 (2014): 859–867, 10.1158/1078-0432.CCR-13-1343. 24334760

[21] M. Prorok‐Hamon , M. K. Friswell , A. Alswied , et al., “Colonic Mucosa‐Associated Diffusely Adherent afaC+ *Escherichia coli* Expressing lpfA and Pks Are Increased in Inflammatory Bowel Disease and Colon Cancer,” Gut 63, no. 5 (2014): 761–770, 10.1136/gutjnl-2013-304739. PMC3995253 23846483

[22] T. W. Battaglia , I. L. Mimpen , J. J. H. Traets , et al., “A Pan‐Cancer Analysis of the Microbiome in Metastatic Cancer,” Cell 187, no. 9 (2024): 2324–2335.e19, 10.1016/j.cell.2024.03.021. 38599211

[23] Y. Jiang , G. Long , X. Huang , W. Wang , B. Cheng , and W. Pan , “Single‐Cell Transcriptomic Analysis Reveals Dynamic Changes in the Liver Microenvironment During Colorectal Cancer Metastatic Progression,” Journal of Translational Medicine 23, no. 1 (2025): 336, 10.1186/s12967-025-06351-3. PMC11910851 40091048

[24] J. Engstrand , H. Nilsson , C. Strömberg , E. Jonas , and J. Freedman , “Colorectal Cancer Liver Metastases—A Population‐Based Study on Incidence, Management and Survival,” BMC Cancer 18, no. 1 (2018): 78, 10.1186/s12885-017-3925-x. PMC5769309 29334918

[25] J. Wirbel , P. T. Pyl , E. Kartal , et al., “Meta‐Analysis of Fecal Metagenomes Reveals Global Microbial Signatures That Are Specific for Colorectal Cancer,” Nature Medicine 25, no. 4 (2019): 679–689, 10.1038/s41591-019-0406-6. PMC7984229 30936547

[26] S. Yachida , S. Mizutani , H. Shiroma , et al., “Metagenomic and Metabolomic Analyses Reveal Distinct Stage‐Specific Phenotypes of the Gut Microbiota in Colorectal Cancer,” Nature Medicine 25, no. 6 (2019): 968–976, 10.1038/s41591-019-0458-7. 31171880

[27] W. Zhang , J. Ling , B. Xu , J. Wang , Z. Chen , and G. Li , “Gut Microbiome‐Mediated Monocytes Promote Liver Metastasis,” International Immunopharmacology 133 (2024): 111877, 10.1016/j.intimp.2024.111877. 38608440

[28] R. Y. C. Hsu , C. H. F. Chan , J. D. Spicer , et al., “LPS‐Induced TLR4 Signaling in Human Colorectal Cancer Cells Increases beta1 Integrin‐Mediated Cell Adhesion and Liver Metastasis,” Cancer Research 71, no. 5 (2011): 1989–1998, 10.1158/0008-5472.CAN-10-2833. 21363926

[29] O. Pabst , M. W. Hornef , F. G. Schaap , V. Cerovic , T. Clavel , and T. Bruns , “Gut–Liver Axis: Barriers and Functional Circuits,” Nature Reviews. Gastroenterology & Hepatology 20, no. 7 (2023): 447–461, 10.1038/s41575-023-00771-6. 37085614

[30] H. Tilg , P. D. Cani , and E. A. Mayer , “Gut Microbiome and Liver Diseases,” Gut 65, no. 12 (2016): 2035–2044, 10.1136/gutjnl-2016-312729. 27802157

[31] H. Zhou , Z. Liu , Y. Wang , et al., “Colorectal Liver Metastasis: Molecular Mechanism and Interventional Therapy,” Signal Transduction and Targeted Therapy 7 (2022): 70, 10.1038/s41392-022-00922-2. PMC8897452 35246503

[32] L. Tian , X. W. Wang , A. K. Wu , et al., “Deciphering Functional Redundancy in the Human Microbiome,” Nature Communications 11 (2020): 6217, 10.1038/s41467-020-19940-1. PMC7719190 33277504

[33] Q. Wang , X. Shen , R. An , et al., “Peritumoral Tertiary Lymphoid Structure and Tumor Stroma Percentage Predict the Prognosis of Patients With Non‐Metastatic Colorectal Cancer,” Frontiers in Immunology 13 (2022): 962056, 10.3389/fimmu.2022.962056. PMC9524924 36189233

[34] Y. Sun , Z. Guo , X. Liu , et al., “Noninvasive Urinary Protein Signatures Associated With Colorectal Cancer Diagnosis and Metastasis,” Nature Communications 13, no. 1 (2022): 2757, 10.1038/s41467-022-30391-8. PMC9119985 35589723

[35] J. Brown , M. Pirrung , and L. A. McCue , “FQC Dashboard: Integrates FastQC Results Into a Web‐Based, Interactive, and Extensible FASTQ Quality Control Tool,” Bioinformatics 33, no. 19 (2017): 3137–3139, 10.1093/bioinformatics/btx373. PMC5870778 28605449

[36] A. M. Bolger , M. Lohse , and B. Usadel , “Trimmomatic: A Flexible Trimmer for ILLUMINA Sequence Data,” Bioinformatics 30, no. 15 (2014): 2114–2120, 10.1093/bioinformatics/btu170. PMC4103590 24695404

[37] K. Rotmistrovsky and R. Agarwala , “BMTagger: Best Match Tagger for Removing Human Reads From Metagenomics Datasets.”

[38] D. E. Wood , J. Lu , and B. Langmead , “Improved Metagenomic Analysis With Kraken 2,” Genome Biology 20, no. 1 (2019): 257, 10.1186/s13059-019-1891-0. PMC6883579 31779668

[39] J. Lu , F. P. Breitwieser , P. Thielen , and S. L. Salzberg , “Bracken: Estimating Species Abundance in Metagenomics Data,” PeerJ Computer Science 3 (2017): e104, 10.7717/peerj-cs.104. PMC12016282 40271438

[40] D. Li , C. M. Liu , R. Luo , K. Sadakane , and T. W. Lam , “MEGAHIT: An Ultra‐Fast Single‐Node Solution for Large and Complex Metagenomics Assembly via Succinct de Bruijn Graph,” Bioinformatics 31, no. 10 (2015): 1674–1676, 10.1093/bioinformatics/btv033. 25609793

[41] D. Hyatt , G. L. Chen , P. F. LoCascio , M. L. Land , F. W. Larimer , and L. J. Hauser , “Prodigal: Prokaryotic Gene Recognition and Translation Initiation Site Identification,” BMC Bioinformatics 11, no. 1 (2010): 119, 10.1186/1471-2105-11-119. PMC2848648 20211023

[42] W. Li and A. Godzik , “Cd‐Hit: A Fast Program for Clustering and Comparing Large Sets of Protein or Nucleotide Sequences,” Bioinformatics 22, no. 13 (2006): 1658–1659, 10.1093/bioinformatics/btl158. 16731699

[43] R. Patro , G. Duggal , M. I. Love , R. A. Irizarry , and C. Kingsford , “Salmon Provides Fast and Bias‐Aware Quantification of Transcript Expression,” Nature Methods 14, no. 4 (2017): 417–419, 10.1038/nmeth.4197. PMC5600148 28263959

[44] B. Buchfink , C. Xie , and D. H. Huson , “Fast and Sensitive Protein Alignment Using DIAMOND,” Nature Methods 12, no. 1 (2015): 59–60, 10.1038/nmeth.3176. 25402007

[45] M. M. Yuan , X. Guo , L. Wu , et al., “Climate Warming Enhances Microbial Network Complexity and Stability,” Nature Climate Change 11, no. 4 (2021): 343–348, 10.1038/s41558-021-00989-9.

[46] R. Guimerà and L. A. Nunes Amaral , “Functional Cartography of Complex Metabolic Networks,” Nature 433, no. 7028 (2005): 895–900, 10.1038/nature03288. PMC2175124 15729348

[47] M. Bastian , S. Heymann , and M. Jacomy , “Gephi: An Open Source Software for Exploring and Manipulating Networks,” Proceedings of the International AAAI Conference on Web and Social Media 3, no. 1 (2009): 361–362, 10.1609/icwsm.v3i1.13937.

[48] A. Liaw and M. Wiener , “Classification and Regression by RandomForest,” R News 2, no. 3 (2002): 18–22.

[49] Q. Feng , S. Liang , H. Jia , et al., “Gut Microbiome Development Along the Colorectal Adenoma–Carcinoma Sequence,” Nature Communications 6, no. 1 (2015): 6528, 10.1038/ncomms7528. 25758642

[50] C. Strobl , A. L. Boulesteix , A. Zeileis , and T. Hothorn , “Bias in Random Forest Variable Importance Measures: Illustrations, Sources and a Solution,” BMC Bioinformatics 8, no. 1 (2007): 25, 10.1186/1471-2105-8-25. PMC1796903 17254353

[51] M. J. Anderson , “A New Method for Non‐Parametric Multivariate Analysis of Variance,” Austral Ecology 26, no. 1 (2001): 32–46, 10.1111/j.1442-9993.2001.01070.pp.x.

[52] I. R. Johannsen , A. K. Boysen , M. F. Nielsen , F. V. Mortensen , and J. Kirkegård , “Liver Metastases and Survival Among Patients With Colorectal Cancer,” JAMA Network Open 8, no. 12 (2025): e2550467, 10.1001/jamanetworkopen.2025.50467. PMC12723549 41632135

[53] A. I. Valderrama‐Treviño , B. Barrera‐Mera , J. C Ceballos‐Villalva , and E. E. Montalvo‐Javé , “Hepatic Metastasis From Colorectal Cancer,” Euroasian Journal of Hepatogastroenterology 7, no. 2 (2017): 166–175, 10.5005/jp-journals-10018-1241. PMC5670263 29201802

[54] E. V. Cutsem , A. Cervantes , R. Adam , et al., “ESMO Consensus Guidelines for the Management of Patients With Metastatic Colorectal Cancer,” Annals of Oncology 27, no. 8 (2016): 1386–1422, 10.1093/annonc/mdw235. 27380959

[55] H. Tilg , T. E. Adolph , R. R. Gerner , and A. R. Moschen , “The Intestinal Microbiota in Colorectal Cancer,” Cancer Cell 33, no. 6 (2018): 954–964, 10.1016/j.ccell.2018.03.004. 29657127

[56] A. M. Patterson , I. E. Mulder , A. J. Travis , et al., “Human Gut Symbiont *Roseburia hominis* Promotes and Regulates Innate Immunity,” Frontiers in Immunology 8 (2017): 8, 10.3389/fimmu.2017.01166. PMC5622956 29018440

[57] F. Rivera‐Chávez , L. F. Zhang , F. Faber , et al., “Depletion of Butyrate‐Producing Clostridia From the Gut Microbiota Drives an Aerobic Luminal Expansion of Salmonella,” Cell Host & Microbe 19, no. 4 (2016): 443–454, 10.1016/j.chom.2016.03.004. PMC4832419 27078066

[58] D. Mouradov , P. Greenfield , S. Li , et al., “Oncomicrobial Community Profiling Identifies Clinicomolecular and Prognostic Subtypes of Colorectal Cancer,” Gastroenterology 165, no. 1 (2023): 104–120, 10.1053/j.gastro.2023.03.205. 36933623

[59] X. Wang , Y. Jia , L. Wen , et al., “ *Porphyromonas gingivalis* Promotes Colorectal Carcinoma by Activating the Hematopoietic NLRP3 Inflammasome,” Cancer Research 81, no. 10 (2021): 2745–2759, 10.1158/0008-5472.CAN-20-3827. 34003774

[60] G. Nakatsu , H. Zhou , W. K. K. Wu , et al., “Alterations in Enteric Virome Are Associated With Colorectal Cancer and Survival Outcomes,” Gastroenterology 155, no. 2 (2018): 529–541.e5, 10.1053/j.gastro.2018.04.018. 29689266

[61] W. S. Garrett , “Cancer and the Microbiota,” Science 348, no. 6230 (2015): 80–86, 10.1126/science.aaa4972. PMC5535753 25838377

[62] D. Ribet and P. Cossart , “Pathogen‐Mediated Posttranslational Modifications: A Re‐Emerging Field,” Cell 143, no. 5 (2010): 694–702, 10.1016/j.cell.2010.11.019. PMC7112265 21111231

[63] E. K. Perry , L. A. Meirelles , and D. K. Newman , “From the Soil to the Clinic: The Impact of Microbial Secondary Metabolites on Antibiotic Tolerance and Resistance,” Nature Reviews. Microbiology 20, no. 3 (2022): 129–142, 10.1038/s41579-021-00620-w. PMC8857043 34531577

[64] C. Lyu , Z. Wang , R. Zhao , et al., “Preoperative Gut Microbial Network Alterations and BCAA‐Related Metabolic Disturbance in Postoperative Delirium After Cardiac Surgery: A Prospective Matched Multi‐Omic Study,” Translational Psychiatry 16, no. 1 (2026): 419, 10.1038/s41398-026-04161-9. PMC13486744 42285959

[65] K. T. Kajihara and N. A. Hynson , “Networks as Tools for Defining Emergent Properties of Microbiomes and Their Stability,” Microbiome 12, no. 1 (2024): 184, 10.1186/s40168-024-01868-z. PMC11439251 39342398

[66] P. Cai , J. Xiong , H. Sha , X. Dai , and J. Lu , “Tumor Bacterial Markers Diagnose the Initiation and Four Stages of Colorectal Cancer,” Frontiers in Cellular and Infection Microbiology 13 (2023): 13, 10.3389/fcimb.2023.1123544. PMC10040638 36992683

[67] A. Gupta , D. B. Dhakan , A. Maji , et al., “Association of *Flavonifractor plautii* , a Flavonoid‐Degrading Bacterium, With the Gut Microbiome of Colorectal Cancer Patients in India,” mSystems 4 (2019): e00438‐19, 10.1128/msystems.00438-19. PMC7407896 31719139

[68] Y. M. Ren , Z. Y. Zhuang , Y. H. Xie , et al., “BCAA‐Producing *Clostridium symbiosum* Promotes Colorectal Tumorigenesis Through the Modulation of Host Cholesterol Metabolism,” Cell Host & Microbe 32, no. 9 (2024): 1519–1535.e7, 10.1016/j.chom.2024.07.012. 39106870

[69] A. V. Robinson and E. Allen‐Vercoe , “Strain Specificity in Fusobacterial Co‐Aggregation With Colorectal Cancer‐Relevant Species,” Anaerobe 82 (2023): 102758, 10.1016/j.anaerobe.2023.102758. 37423597

[70] S. Liu , W. Zhao , P. Lan , and X. Mou , “The Microbiome in Inflammatory Bowel Diseases: From Pathogenesis to Therapy,” Protein & Cell 12, no. 5 (2021): 331–345, 10.1007/s13238-020-00745-3. PMC8106558 32601832

[71] M. Vital , A. C. Howe , and J. M. Tiedje , “Revealing the Bacterial Butyrate Synthesis Pathways by Analyzing (Meta)genomic Data,” MBio 5, no. 2 (2014): e00889‐14, 10.1128/mBio.00889-14. PMC3994512 24757212

[72] G. Zeller , J. Tap , A. Y. Voigt , et al., “Potential of Fecal Microbiota for Early‐Stage Detection of Colorectal Cancer,” Molecular Systems Biology 10, no. 11 (2014): 766, 10.15252/msb.20145645. PMC4299606 25432777

[73] D. Maclean , M. Tsakok , F. Gleeson , et al., “Comprehensive Imaging Characterization of Colorectal Liver Metastases,” Frontiers in Oncology 11 (2021): 730854, 10.3389/fonc.2021.730854. PMC8688250 34950575

[74] J. H. Lee and S. W. Lee , “The Roles of Carcinoembryonic Antigen in Liver Metastasis and Therapeutic Approaches,” Gastroenterology Research and Practice 2017 (2017): 7521987, 10.1155/2017/7521987. PMC5447280 28588612

[75] M. Renzulli , A. Clemente , A. M. Ierardi , et al., “Imaging of Colorectal Liver Metastases: New Developments and Pending Issues,” Cancers (Basel) 12, no. 1 (2020): 151, 10.3390/cancers12010151. PMC7017094 31936319

[76] B. D. Nicholson , B. Shinkins , I. Pathiraja , et al., “Blood CEA Levels for Detecting Recurrent Colorectal Cancer,” Cochrane Database of Systematic Reviews 2015, no. 12 (2015): CD011134, 10.1002/14651858.CD011134.pub2. PMC7092609 26661580

[77] I. Mignini , G. Piccirilli , L. Galasso , et al., “From the Colon to the Liver: How Gut Microbiota May Influence Colorectal Cancer Metastatic Potential,” Journal of Clinical Medicine 13, no. 2 (2024): 420, 10.3390/jcm13020420. PMC10815973 38256554

[78] C. K. Y. Lau , K. D. Krewulak , and H. J. Vogel , “Bacterial Ferrous Iron Transport: The Feo System,” FEMS Microbiology Reviews 40, no. 2 (2016): 273–298, 10.1093/femsre/fuv049. 26684538

[79] M. Ellermann and J. C. Arthur , “Siderophore‐Mediated Iron Acquisition and Modulation of Host‐Bacterial Interactions,” Free Radical Biology and Medicine 105 (2017): 68–78, 10.1016/j.freeradbiomed.2016.10.489. PMC5401654 27780750

