# Supplementary material for: The Potential Role of Gut Microbiome Dysbiosis and Functional Reprogramming in Colorectal Liver Metastasis via the Gut–Liver Axis
Source: Cancer Rep (Hoboken). 2026 Nov 4;9(11):e70738. doi: 10.1002/cnr2.70738 (PMC13732294; doi:10.1002/cnr2.70738)
Supplement: Supplementary file 1 [file CNR2-9-e70738-s001.doc]

**Table S1** Demographic and clinical characteristics in the CON, CRC, and CRLM groups.

| Items | CON (n = 20) | CRC (n = 20) | CRLM (n = 20) |
| --- | --- | --- | --- |
| Gender (Male/Female) | 10/10 | 11/9 | 10/10 |
| Age | 44.15 ± 10.399^b^ | 59.00 ± 9.274^a^ | 63.85 ± 6.839^a^ |
| BMI (kg/m^2^) | 20.96 ± 1.587^c^ | 22.95 ± 1.141^b^ | 28.16 ± 1.072^a^ |
| Tumor type | | | |
| N/A | 20 | 0 | 0 |
| Adenocarcinoma | 0 | 20 | 20 |
| Tumor stage | | | |
| N/A | 20 | 0 | 0 |
| Ⅰ | 0 | 3 | 0 |
| Ⅱ | 0 | 5 | 0 |
| Ⅲ | 0 | 5 | 0 |
| Ⅳ | 0 | 7 | 20 |
| Tumor site | | | |
| N/A | 20 | 0 | 0 |
| Rectum | 0 | 10 | 10 |
| Colon | 0 | 10 | 10 |

**Table S2.** Raw and clean sequencing yields for individual samples.

| Sample ID | Group | Raw sequence yield (Gb) | Clean sequence yield (Gb) |
| --- | --- | --- | --- |
| CON1 | CON | 10 | 6.09 |
| CON2 | CON | 10 | 6.83 |
| CON3 | CON | 10 | 6.28 |
| CON4 | CON | 10 | 6.68 |
| CON5 | CON | 10 | 5.73 |
| CON6 | CON | 10 | 6.46 |
| CON7 | CON | 10 | 6.75 |
| CON8 | CON | 10 | 6.13 |
| CON9 | CON | 10 | 6.57 |
| CON10 | CON | 10 | 5.95 |
| CON11 | CON | 10 | 6.97 |
| CON12 | CON | 10 | 6.24 |
| CON13 | CON | 10 | 6.53 |
| CON14 | CON | 10 | 5.87 |
| CON15 | CON | 10 | 6.61 |
| CON16 | CON | 10 | 6.33 |
| CON17 | CON | 10 | 6.42 |
| CON18 | CON | 10 | 6.02 |
| CON19 | CON | 10 | 6.37 |
| CON20 | CON | 10 | 6.17 |
| CRC1 | CRC | 10 | 6.27 |
| CRC2 | CRC | 10 | 5.72 |
| CRC3 | CRC | 10 | 6.68 |
| CRC4 | CRC | 10 | 5.96 |
| CRC5 | CRC | 10 | 6.44 |
| CRC6 | CRC | 10 | 5.48 |
| CRC7 | CRC | 10 | 6.22 |
| CRC8 | CRC | 10 | 6.58 |
| CRC9 | CRC | 10 | 5.86 |
| CRC10 | CRC | 10 | 6.82 |
| CRC11 | CRC | 10 | 6.13 |
| CRC12 | CRC | 10 | 6.39 |
| CRC13 | CRC | 10 | 5.62 |
| CRC14 | CRC | 10 | 6.17 |
| CRC15 | CRC | 10 | 6.51 |
| CRC16 | CRC | 10 | 6.03 |
| CRC17 | CRC | 10 | 5.79 |
| CRC18 | CRC | 10 | 6.34 |
| CRC19 | CRC | 10 | 6.08 |
| CRC20 | CRC | 10 | 5.91 |
| CRLM1 | CRLM | 10 | 6.04 |
| CRLM2 | CRLM | 10 | 6.73 |
| CRLM3 | CRLM | 10 | 6.32 |
| CRLM4 | CRLM | 10 | 7.19 |
| CRLM5 | CRLM | 10 | 6.14 |
| CRLM6 | CRLM | 10 | 6.83 |
| CRLM7 | CRLM | 10 | 6.48 |
| CRLM8 | CRLM | 10 | 6.96 |
| CRLM9 | CRLM | 10 | 5.91 |
| CRLM10 | CRLM | 10 | 6.62 |
| CRLM11 | CRLM | 10 | 6.89 |
| CRLM12 | CRLM | 10 | 6.37 |
| CRLM13 | CRLM | 10 | 7.06 |
| CRLM14 | CRLM | 10 | 6.21 |
| CRLM15 | CRLM | 10 | 6.78 |
| CRLM16 | CRLM | 10 | 6.53 |
| CRLM17 | CRLM | 10 | 6.67 |
| CRLM18 | CRLM | 10 | 6.27 |
| CRLM19 | CRLM | 10 | 6.57 |
| CRLM20 | CRLM | 10 | 6.43 |

**Table S3** Pairwise Adonis (PERMANOVA) analysis of gut microbial community structure among the CON, CRC, and CRLM groups.

| Comparison | *F* value | *R²* | *P* value |
| --- | --- | --- | --- |
| CON vs. CRC | 1.685 | 0.042 | 0.088 |
| CON vs. CRLM | 3.928 | 0.094 | 0.001 |
| CRC vs. CRLM | 3.228 | 0.078 | 0.005 |

**Table S4** Comparison of significantly different KEGG pathways among the CON, CRC, and CRLM groups.

| **Pathways** | *P* value (CRC vs CON) | *P* value (CRLM vs CON) | *P* value (CRLM vs CRC) |
| --- | --- | --- | --- |
| Glycerolipid metabolism | **0.001046** | **0.008213** | — |
| Citrate cycle (TCA cycle) | **0.002190** | **0.023241** | — |
| Carbon metabolism | **0.004997** | **0.039640** | — |
| Virion-Bacteriophage lambda | 0.036148 | **0.006977** | **0.003886** |
| Glyoxylate and dicarboxylate metabolism | **0.045795** | **0.007591** | **0.024543** |
| Bacterial secretion system | **0.007637** | **0.016475** | — |
| Cationic antimicrobial peptide (CAMP) resistance | **0.023908** | **0.008180** | **0.041836** |
| Glycine, serine and threonine metabolism | **0.018102** | **0.013058** | — |
| O-Antigen nucleotide sugar biosynthesis | — | **0.013797** | **0.019341** |
| Biosynthesis of nucleotide sugars | — | **0.017883** | **0.005445** |
| Yersinia infection | 0.008258 | **0.020603** | **0.024094** |
| Teichoic acid biosynthesis | **0.029039** | **0.020632** | — |
| Biosynthesis of siderophore group nonribosomal peptides | — | **0.022232** | **0.020163** |
| Biosynthesis of various antibiotics | — | **0.027581** | **0.008311** |
| Monobactam biosynthesis | — | **0.032343** | — |
| Antifolate resistance | — | **0.033179** | **0.044996** |
| Lipopolysaccharide biosynthesis | **0.035038** | — | — |
| Methane metabolism | **0.022374** | **0.037492** | — |
| Biotin metabolism | **0.047520** | — | — |
| Carbon fixation pathways in prokaryotes | **0.048727** | — | — |

Table S5 Module hubs and connectors in the CON microbial co-occurrence network.

| Taxon | Within_module_connectivities | Among_module_connectivities | Topological role |
| --- | --- | --- | --- |
| *Fusobacterium pseudoperiodonticum* | 2.740820456 | 0 | Module hubs |
| *Mogibacterium diversum* | 2.773724642 | 0 | Module hubs |
| *Bacteroides xylanisolvens* | 3.049719894 | 0 | Module hubs |
| *Butyricimonas faecalis* | -1.458450282 | 0.625 | Connectors |
| *Lachnoanaerobaculum gingivalis* | -0.119066491 | 0.693877551 | Connectors |
| *Clostridioides difficile* | -0.119066491 | 0.666666667 | Connectors |
| *Mediterraneibacter gnavus* | -0.754087776 | 0.625 | Connectors |
| *Parabacteroides absconsus* | -0.754087776 | 0.625 | Connectors |
| *Roseburia sp. 831b* | -1.389109061 | 0.666666667 | Connectors |
| *Parabacteroides distasonis* | -0.257205292 | 0.7 | Connectors |
| *Simiaoa sunii* | -0.257205292 | 0.65625 | Connectors |
| *Bacteroides thetaiotaomicron* | -0.257205292 | 0.693877551 | Connectors |
| *Streptococcus salivarius* | -0.80835949 | 0.64 | Connectors |
| *Anaerostipes hadrus* | -0.80835949 | 0.625 | Connectors |
| *Clostridium sp.* M62/1 | 0.304535606 | 0.65625 | Connectors |
| *Blautia luti* | -0.913606819 | 0.722222222 | Connectors |
| *Schaalia odontolytica* | -0.913606819 | 0.625 | Connectors |
| *Eikenella corrodens* | -0.781511729 | 0.64 | Connectors |
| *Dorea longicatena* | -1.598546719 | 0.666666667 | Connectors |
| *Enterocloster bolteae* | -1.598546719 | 0.666666667 | Connectors |
| *Faecalimonas umbilicata* | 0 | 0.666666667 | Connectors |
| *Dialister pneumosintes* | 0 | 0.666666667 | Connectors |
| *Phocaeicola vulgatus* | -0.924574881 | 0.72 | Connectors |
| *Dysosmobacter welbionis* | -0.924574881 | 0.625 | Connectors |
| *Flintibacter sp.* KGMB00164 | -0.924574881 | 0.625 | Connectors |
| *Candidatus Nanosynbacter sp.* HMT-352 | -0.924574881 | 0.625 | Connectors |
| *Anaerotruncus colihominis* | -1.849149761 | 0.666666667 | Connectors |

**Table S6** Module hubs and connectors in the CRC microbial co-occurrence network.

| Taxon | Within_module_connectivities | Among_module_connectivities | Topological role |
| --- | --- | --- | --- |
| *Odoribacter splanchnicus* | 2.826635489 | 0 | Module hubs |
| *Bacteroides sp.* A1C1 | 2.848579116 | 0 | Module hubs |
| *Phocaeicola salanitronis* | 2.848579116 | 0 | Module hubs |
| *Hoylesella buccalis* | -0.39672077 | 0.666666667 | Connectors |
| *Roseburia hominis* | -0.39672077 | 0.666666667 | Connectors |
| *Faecalibacterium sp.* I3-3-33 | -1.041392022 | 0.64 | Connectors |
| *[Ruminococcus] lactaris* | -1.041392022 | 0.625 | Connectors |
| *Alistipes onderdonkii* | -1.164274327 | 0.666666667 | Connectors |
| *Enterocloster asparagiformis* | -0.437089704 | 0.734693878 | Connectors |
| *Pseudoflavonifractor gallinarum* | -0.437089704 | 0.666666667 | Connectors |
| *Faecalibacillus intestinalis* | -1.012207735 | 0.72 | Connectors |
| *Bacteroides finegoldii* | -1.012207735 | 0.72 | Connectors |
| *Lawsonibacter asaccharolyticus* | -1.012207735 | 0.64 | Connectors |
| *Enterocloster lavalensis* | -1.012207735 | 0.64 | Connectors |
| *Eubacterium ramulus* | -1.012207735 | 0.625 | Connectors |
| *Roseburia sp.* 831b | 0.188398055 | 0.653061224 | Connectors |
| *Eubacterium callanderi* | -1.318786386 | 0.666666667 | Connectors |
| *Bacteroides salyersiae* | -0.565194165 | 0.625 | Connectors |
| *Coprococcus sp.* ART55/1 | -1.318786386 | 0.75 | Connectors |
| *Bacteroides nordii* | -1.318786386 | 0.666666667 | Connectors |
| *Flintibacter sp.* KGMB00164 | -1.318786386 | 0.666666667 | Connectors |
| *Bacteroides sp.* CG01 | 0.175823714 | 0.691358025 | Connectors |
| *Blautia parvula* | -0.527471143 | 0.693877551 | Connectors |
| *Blautia hydrogenotrophica* | -1.230766 | 0.64 | Connectors |
| *Faecalibacterium prausnitzii* | 0.593012753 | 0.641975309 | Connectors |
| *Pusillibacter faecalis* | -0.158842702 | 0.653061224 | Connectors |
| *Prevotella denticola* | -0.158842702 | 0.734693878 | Connectors |
| *Enterocloster bolteae* | -0.910698156 | 0.64 | Connectors |
| *Bacteroides xylanisolvens* | -0.910698156 | 0.625 | Connectors |
| *Bacteroides intestinalis* | -0.910698156 | 0.625 | Connectors |
| *Faecalibacterium sp.* I2-3-92 | -0.910698156 | 0.625 | Connectors |
| *Flavonifractor plautii* | -1.66255361 | 0.666666667 | Connectors |
| *Eggerthella lenta* | -1.66255361 | 0.666666667 | Connectors |
| *Ruminococcus sp.* SR1/5 | -1.66255361 | 0.666666667 | Connectors |

**Table S7** Module hubs and connectors in the CRLM microbial co-occurrence network.

| Taxon | Within_module_connectivities | Among_module_connectivities | Topological role |
| --- | --- | --- | --- |
| *Campylobacter concisus* | 2.65628219 | 0.21875 | Module hubs |
| *Coprococcus eutactus* | 2.557094638 | 0.375 | Module hubs |
| *[Clostridium] symbiosum* | 2.557094638 | 0 | Module hubs |
| *Phocaeicola salanitronis* | -1.507619621 | 0.666666667 | Connectors |
| *Eubacterium ventriosum* | -1.507619621 | 0.666666667 | Connectors |
| *Faecalimonas umbilicata* | -1.022199821 | 0.625 | Connectors |
| *Bacteroides sp.* M10 | -1.022199821 | 0.625 | Connectors |
| *Lachnospira eligens* | 0.889020605 | 0.625 | Connectors |
| *Dorea longicatena* | -0.977922666 | 0.64 | Connectors |
| *Dorea formicigenerans* | -0.977922666 | 0.625 | Connectors |
| *Enterocloster lavalensis* | -0.977922666 | 0.625 | Connectors |
| *Parabacteroides absconsus* | -0.977922666 | 0.625 | Connectors |
| *Bacteroides faecis* | -0.525172783 | 0.625 | Connectors |
| *Parabacteroides faecis* | -0.958910489 | 0.625 | Connectors |
| *Roseburia faecis* | -1.837911771 | 0.666666667 | Connectors |

**Table S8** Abundance comparison of the top 10 features selected by the random forest model between the CRC and CRLM groups.

| **Marker** | **Feature type** | ***P* value** |
| --- | --- | --- |
| *Staphylococcus* | genus | 0.047221 |
| *Corynebacterium* | genus | 0.001441 |
| *Finegoldia* | genus | 0.001225 |
| *Neisseria* | genus | 0.008706 |
| *Hoylesella* | genus | 0.049382 |
| *Gemella* | genus | 0.529286 |
| *Anaerococcus* | genus | 0.000687 |
| *Pseudomonas* | genus | 0.563542 |
| VF0160 *FeoAB* | virulence factor | 0.252437 |
| *Eggerthella* | genus | 0.369492 |

**Figure S1** Random Forest (RF) model based on microbiome for distinguishing CRC from CON. The horizontal bar plot displays the Top 10 features ranked by the percentage of Mean Decrease Accuracy (%). The inset illustrates the feature selection process based on nested cross-validation, showing the variation in inner cross-validation error relative to the number of features, with the dashed line indicating the point of minimum error (a). The scatter plot illustrates the predicted probabilities of the model for the samples (b). The horizontal dashed line denotes the classification threshold at a predicted result of 0.50. Data points are color-coded according to the predicted category: red (TRUE) represents a prediction of CRLM, while green (FALSE) represents a prediction of CRC.

**Figure S2** RF model based on microbiome for distinguishing CRLM from CON. Figure legend is similar to Supplementary Figure S1.

**Figure S3** RF model based on microbiome for distinguishing CRLM from CRC. Figure legend is similar to Supplementary Figure S1.

Figure S4 RF model based on virulence factors for distinguishing CRC from CON. Figure legend is similar to Supplementary Figure S1.

Figure S5 RF model based on virulence factors for distinguishing CRLM from CON. Figure legend is similar to Supplementary Figure S1.

Figure S6 RF model based on virulence factors for distinguishing CRLM from CRC. Figure legend is similar to Supplementary Figure S1.

Figure S7 RF model integrating microbiome and virulence factors for distinguishing CRC from CON. Figure legend is similar to Supplementary Figure S1.

Figure S8 RF model integrating microbiome and virulence factors for distinguishing CRLM from CON. Figure legend is similar to Supplementary Figure S1.
